# Nuclear pore passage of the HIV capsid is driven by its unusual surface amino acid composition

**DOI:** 10.1038/s41594-025-01684-5

**Published:** 2025-10-09

**Authors:** Liran Fu, Shiya Cheng, Dietmar Riedel, Leonie Kopecny, Melina Schuh, Dirk Görlich

**Affiliations:** 1 Department of Cellular Logistics, Max Planck Institute for Multidisciplinary Sciences, Göttingen, Germany; 2 Department of Meiosis, Max Planck Institute for Multidisciplinary Sciences, Göttingen, Germany; 3 Laboratory for Electron Microscopy, Max Planck Institute for Multidisciplinary Sciences, Göttingen, Germany; 4 Present Address: School of Basic Medical Sciences, Wuhan University, Wuhan, China

**Keywords:** Nuclear pore complex, Pathogens, Virology, Molecular biology

## Abstract

Nuclear transport receptors (NTRs) carry cargo across the permeability barrier of nuclear pore complexes (NPCs)—an FG phase condensed from disordered but cohesive FG-repeat domains. This phase repels inert macromolecules but allows NTR passage. When the human immunodeficiency virus (HIV) infects nondividing cells, its capsid is transported into nuclei not like a cargo but crosses NPCs like an NTR. Here we uncovered the molecular determinants of the capsid’s NTR behavior. The FG-binding pocket is insufficient. Hexameric and pentameric capsomers contribute. The highly exposed outer capsid surface is key. It lacks FG-repulsive charged residues (K, D and E) that are very abundant on other protein surfaces. FG-attractive residues dominate the capsid surface instead. Introducing FG-repulsive amino acids impedes FG phase partitioning, NPC targeting and NPC passage of assembled capsids. Capsids are, thus, made soluble in the FG phase by a myriad of transient FG-attractive interactions originating from individual surface side chains. We propose that CPSF6 releases the capsid from NPCs by masking its FG-attractive surface and switching the capsid to an FG-repulsive species.

## Abstract (web summary)

Fu et al. uncover the features that allow the HIV capsid to cross the permeability barrier of nuclear pores in an importin-independent manner, explaining how the viral genome can reach the nuclear compartment when infecting nondividing cells.

## Main

Nuclear pore complexes (NPCs) are embedded in the nuclear envelope (NE) to conduct nucleocytoplasmic transport^1–3^. This includes receptor-mediated import of nuclear proteins and export of mRNAs or newly assembled ribosomes^4–6^. NPCs are equipped with a barrier that can be rationalized as an FG phase^7–10^, assembled from intrinsically disordered but cohesive FG-repeat domains. This barrier is selectively permeable to nuclear transport receptors (NTRs) along with captured transport substrates while rejecting macromolecules that are not recognized as valid cargo.

Typical NPCs contain ~12 different FG-repeat domains anchored through distinct nucleoporins (Nups). Of these, the FG domain of Nup98 (refs. ^11,12^) appears to be the most critical one for the NPC barrier^13^. It occurs in high copy number^14^, is highly depleted of charged residues, is poorly soluble in water and, therefore, readily phase-separates from aqueous solutions^8^. The resulting condensed FG phase shows essentially the same permeability properties as NPCs themselves^8,15^. It excludes inert macromolecules (such as mCherry) but dissolves NTRs to high partition coefficients. NTRs recognize and bind cargoes^4–6^. They promote barrier passage of a selected cargo by enhancing its solubility within the FG phase^7^. The phase behavior of ‘cohesive’ FG-repeat domains^8,13,16–24^ and an FG phase-related transport selectivity^7,8,13,18,25–27^ have been documented in a range of experimental systems. There is genetic evidence from yeast^16,20^ and striking evolutionary evidence^8^ for a cohesive permeability barrier. Furthermore, atomic force microscopy was used to detect a gel-like material at NPCs^28^; it has been reported that mild interference with hydrophobic (FG) interactions by hexanediols leads to a reversible collapse of the permeability barrier^29,30^ and NPCs become nonselectively permeable when the cohesive Nup98 FG domain is replaced by domains that bind NTRs but are noncohesive^13^.

The importin-β superfamily represents the largest NTR class^4,31,32^ and includes nuclear import receptors (importins), exportins and biportins. Asymmetric transport cycles driven by the RanGTPase system^33^ allow an active pumping of cargoes against a concentration gradient. Importins, for example, capture cargo in the cytoplasm, translocate through NPCs and release cargo into the nucleus upon RanGTP binding. While the permeability barrier retains imported cargo inside nuclei, importin–RanGTP complexes return to the cytoplasm, where GTP hydrolysis releases Ran and allows the importins to import the next cargo molecule.

Importin-β NTRs are 90–140-kDa α-solenoid proteins^32^. Other NTRs include NTF2 (the importer of Ran)^34^, the Mex67–Mtr2 dimer (the main exporter of RNA)^35,36^ and Hikeshi (the importer of Hsp70)^37^. All NTRs bind FG repeats in a multivalent fashion^36,38–40^. This binding counteracts the hydrophobic cohesive interactions among repeats^41^ and, thus, allows an NTR to ‘melt’ through the FG phase^42^. It was recently discovered that the human immunodeficiency virus 1 (HIV-1) capsid also functions as an NTR^43,44^.

HIV is the retrovirus that causes AIDS (acquired immunodeficiency syndrome) by infecting and eventually eliminating immune cells^45–47^. Its capsid initially encloses two copies of the HIV genomic RNA^48^, along with reverse transcriptase and integrase^49,50^. It is enveloped by a viral membrane that fuses with the plasma membrane during entry into a target cell^51^. This membrane fusion releases the capsid into the cytoplasm, where an influx of deoxynucleotide triphosphates into the capsid initiates reverse transcription^52^. The resulting vDNA is eventually integrated into a chromosome^53^. Simple retroviruses require (the open) mitosis to access chromatin. HIV and other lentiviruses, however, efficiently infect nondividing cells with intact NEs^54^, suggesting that an incoming HIV genome can somehow cross NPCs.

It was long believed that the viral capsid (60 nm × 120 nm) was too large for NPC passage, that capsid uncoating and release of the HIV genome occurred in the cytoplasm and that the capsid-free preintegration complex was the species imported into the cell nucleus (discussed in ref. ^55^). This view was recently revised when intact capsids were detected inside nuclei during the course of infection^56–60^, when cryo-EM tomograms showed incoming capsids trapped at NPCs^61^ and when the diameter of the NPC scaffold was shown to be larger in intact cells (~60 nm)^62^ than in the specimens earlier analyzed from isolated NEs^63^. Lastly, it was discovered that mature capsids behave like NTRs, efficiently partitioning into an FG phase and targeting NPCs without any *trans*-acting factors^43,44^. This NTR-like behavior required capsid assembly. These findings also resolved the problem that the width of the NPC scaffold (60 nm) is just sufficient to accommodate a ‘naked’ but not importin-coated capsid.

CPSF6 (cleavage and polyadenylation specificity factor 6) is a host factor with a nuclear localization involved in capsid transport^64–66^. It contains a single, rather unusual FG ‘repeat’ that can dock into a pocket of the capsid^67,68^. It releases capsids from NPCs into the nucleoplasm and guides them to prospective chromosomal integration sites^56,57^.

Capsid assembly^69^ begins with translation of the gag polyprotein (reviewed in ref. ^70^), which comprises the MA (matrix protein), CA (p24 capsid protein), NC (nucleocapsid) and p6 modules. MA gets myristoylated and targets gag to the plasma membrane^71,72^. CA assembles into the immature capsid^73^ in an IP6 (d-*myo*-inositol hexakisphosphate)-assisted manner^74^, whereas NC recruits HIV genomic RNA to the nascent virions^75^. P6 initiates virus budding from the plasma membrane^76^. The switch to mature capsids is triggered by the HIV protease^77^, which cleaves gag into its individual proteins^78^. This switch involves major rearrangements in the capsomer structures^79^. This elaborate assembly sequence probably prevents the capsid from getting (mis)targeted to NPCs already in virus-producing cells.

## Results

### Not only CA hexamers but also pentamers confer an efficient FG barrier entry

This study focuses on molecular features that allow mature HIV-1 capsids to pass the FG barrier of NPCs and, thus, deliver the viral genome into the nucleus. This passage should rely on interactions with FG repeats. Indeed a deep binding pocket between the subunits of the CA hexamers has been described, which can accommodate the unique FG repeat of CPSF6 or a Nup153 FG repeat^66–68^. There, the side-chain amide of N57 of the CA protein engages in a double hydrogen bond with the backbone of the FG peptide. The N57A mutation abrogates this stable binding^66,80,81^ and impedes NPC passage^43^ and the partitioning into a Nup98 FG phase (refs. ^43,44^ and figures below). The capsid comprises not only hexamers but also pentameric capsomers^82^. Pentamers are thought not to bind FG repeats; a lack of CPSF6 FG binding was documented and explained by an N57 pocket rearrangement^83,84^.

**Fig. 1:**
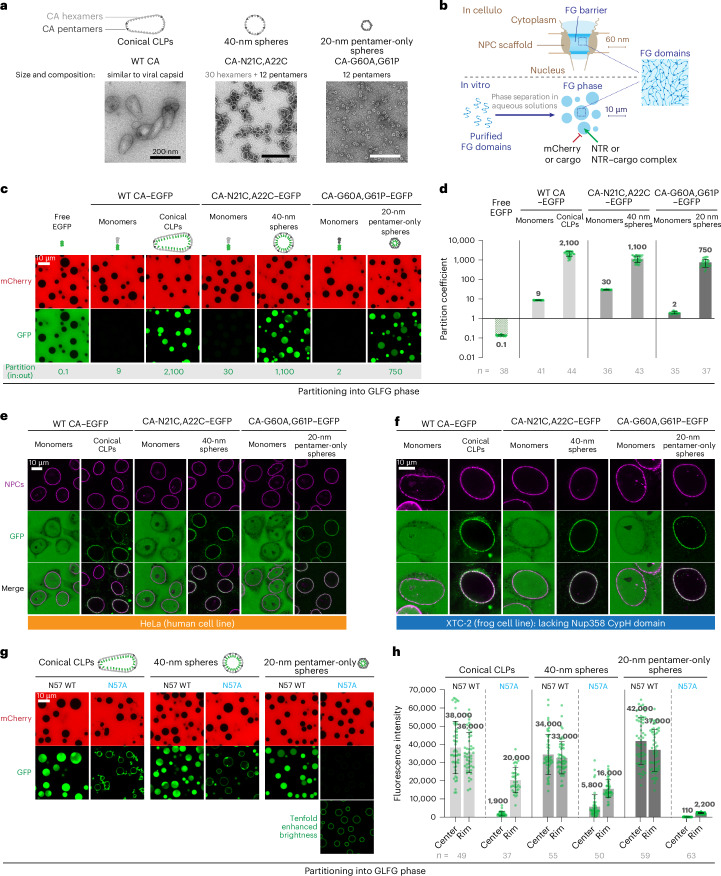
Hexameric and pentameric capsomers mediate FG phase partitioning and NPC targeting. **a**, Negative-stain electron micrographs of the HIV-1 capsid species used. WT, wild type. **b**, Concept of the FG phase as a permeability barrier of NPCs, reconstitution of such a barrier by phase separation of barrier-forming cohesive FG domains and testing of its transport properties. **c**, A Nup98-type FG domain comprising 52 perfect 12-mer GLFG repeat units (prf.GLFG_52x12_)^88^ was allowed to phase separate, forming FG phases of near-spherical shape. Indicated fluorescent species were subsequently added and detected 1 h later by confocal laser scanning microscopy (CLSM). Free mCherry and EGFP remained well excluded from the FG phase, with partition coefficients (in:out) of ≤0.1. Large CLPs, 40-nm spheres and 20-nm T1 ‘pentamer-only’ spheres were each labeled by ~15% CA with a C-terminally fused EGFP, pointing to the capsids’ interior. They accumulated essentially completely inside the FG phase, reaching partition coefficients of ~1,000. This was 30–300 times higher than the corresponding monomeric CA–EGFP fusions and 10,000 times higher than free EGFP. **d**, Partition coefficients of EGFP, indicated CA monomers or capsid species into the FG phase were calculated by dividing the signals inside FG particles by outside signals. Numbers are means; bars indicate the mean ± s.d. (*n* = number of quantified FG particles). **e**,**f**, Specific targeting of capsid species to NPCs. Human HeLa cells or frog XTC-2 cells (whose Nup358 lacks a CypH domain) were grown on coverslips, treated with digitonin to permeabilize their plasma membranes and incubated with the indicated fluorescent species. Confocal scans were taken directly through the live samples 30 min later. Laser settings were individually adjusted. NPCs were detected with an Alexa647-labeled anti-Nup133 nanobody^89^. Note that conical CLPs, 40-nm capsid spheres and pentamer-only spheres showed highly efficient NPC targeting in both cell types, comparable to the nanobody staining. By contrast, unassembled CA monomers showed no NPC enrichment and were evenly distributed throughout the cells. **g**,**h**, FG phase partitioning of all three capsid species is strongly reduced by the N57A mutation. Experiments and quantifications were performed as in **c**,**d** but signals at the phase surface (rim) and the center are shown separately (see also below, Fig. 3b,c) (*n* = number of quantified FG particles). Tabular data, including *t*-test *P* values for statistical significance of group differences, are provided in Supplementary Data 1. Scan settings were identical for each wild type–N57A mutant pair. Scale bars, 200 nm (**a**) and 10 μm (**c**,**e**–**g**). Experiments were independently replicated four times, yielding consistent outcomes. Source data

Nevertheless, we wondered whether pentamers contribute to FG phase entry. To investigate this, we used the CA-G60A+G61P double mutant to reconstitute 20-nm T1 capsid spheres, built solely from 12 pentamers^83^. For comparison, we also assembled capsids from wild-type CA^83,85^, yielding large (~80 × 160 nm) cone-shaped CLPs (capsid-like particles) dominated by hexamers, as well as 40-nm capsid spheres with 30 hexamers and 12 pentamers^86,87^. These capsid species were all labeled by 15% GFP fusion to the CA C terminus that points to the capsid’s interior. Negative-stain electron microscopy (EM) confirmed proper assembly (Fig. 1a).

**Extended Data Fig. 1:**
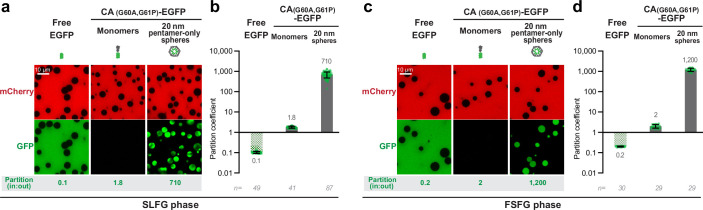
Pentamer-only capsids partition efficiently into SLFG and FSFG phases. FG phase-partitioning experiments and quantifications were performed as in Fig. 1c-d, but FG phases were assembled from different FG repeat domains. Note that fully assembled T1 spheres partitioned very well also in these alternative FG phases – 500 times better than the unassembled CA monomers. Partition coefficients of EGFP, indicated CA monomers or capsids into the FG phase were calculated as in Fig. 1d. Numbers are means; bars indicate mean ± s.d.; *n* = number of quantified FG particles. See Supplementary Excel file for tabular data, including Student’s *t*-test *P* values for statistical significance of group differences. Scan settings were identical for each monomer-capsid pair. Experiments were independently replicated three times with consistent outcomes. Scale bars, 10 μm. **a,b**, Instead of the 52x GLFG 12mer repeats (G**GLFG**GNTQPAT)_52_ as in Fig. 1, a domain with 52 SLFG 12mer repeat units was used (ref. ^41^; see Supplementary Table S1 for sequence). The inter-FG spacer was also free of charged amino acids – a typical feature of Nup98-type FG repeats. SLFG is the second most abundant FG motif in Nup98 FG repeat domains. **c,d**, An FG repeat domain of 669 residues with non-charged spacers and 36 FSFG motifs was used. The lower motif density (1 motif per 18 residues) considers that FSFG motifs are more hydrophobic and thus more cohesive than GLFG or SLFG motifs. For sequence see ref. ^41^ or Supplementary Table S1. Source data

As a proxy for the NPC barrier, we assembled an FG phase from Nup98-type perfect GLFG 12-mer repeats^88^. CLPs and 40-nm capsid spheres accumulated in this phase to partition coefficients of 1,000–2,000 (Fig. 1c,d), consistent with our previous observations^43^. Surprisingly, the pentamer-only spheres behaved the same, reaching an intraphase partition coefficient of ~750 (Fig. 1c,d). In FG phases with other common FG motifs (SLFG or FSFG), they partitioned to similarly high coefficients (Extended Data Fig. 1). This partitioning was specific; unassembled protomers accumulated 300–600 times less, whereas mCherry (our internal, inert control) or nonfused GFP remained completely excluded (with partition coefficients of ≤0.1).

### NPC targeting of capsids can occur independently of the Nup358 CypH (cyclophilin homology) domain

In complementary assays with digitonin-semipermeabilized HeLa cells, we observed that not only CLPs and 40-nm spheres but also pentamer-only spheres targeted NPCs with very high efficiency and then colocalized with an anti-Nup133 nanobody (Fig. 1e)^89^.

**Extended Data Fig. 2:**
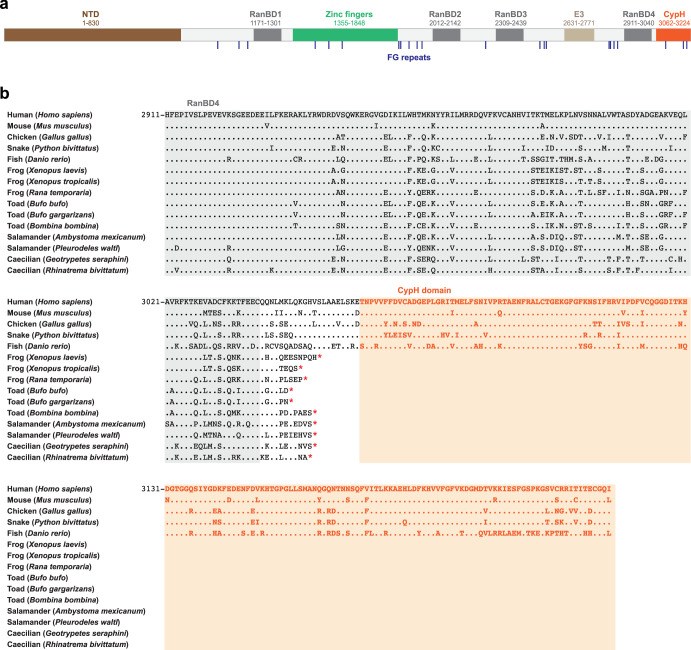
*Xenopus* Nup358/ RanBP2 lacks the C-terminal CypH (cyclophilin homology) domain. **a**, Domain organization of human Nup358, which includes an N-terminal NPC-anchor domain, four RanBP1-like Ran-binding domains (RanBD1-4), 8 Ran-binding zinc finger domains, two SUMO-E3 ligase domains, several FG repeat domains, and a C-terminal CypH domain. **b**, Sequence alignment of the C terminus part of Nup358 from indicated species. Note that Nup358 from mammals, birds, reptiles, or fish all have a C-terminal CypH domain. However, the CypH domain has been lost in modern amphibians such as frogs, toads, salamanders, and caecilians, where a translation stop is found immediately after the last RanBD. Cells from the frog/ toad *Xenopus laevis* can therefore be considered as a naturally occurring ΔCypH mutant. Sequence identifiers are listed in Supplementary Table S6.

NPC targeting could be mediated by direct FG interactions and/or by the CypH domain of Nup358/RanBP2, which binds the cyclophilin-binding loop (loop 2) of the capsid^90^. To disentangle these potential contributions, we repeated the NPC-targeting experiment using the XTC-2 cell line^91^ from the frog *Xenopus laevis*. Although frogs (like all modern amphibians) have lost the CypH domain from their Nup358 (Extended Data Fig. 2), we again observed efficient capsid targeting to NPCs (Fig. 1f). This indicates a highly efficient capsid entry into the FG phase of NPCs. This was also true for the pentamer-only capsids, confirming that CA pentamers mediate productive FG interactions. The experiment also showed that the capsid–FG phase interactions are independent of species-specific FG domain features.

### Crucial FG interactions by the CA pentamers’ N57 pocket

The rearrangement of the N57 FG-binding pocket in CA pentamers^83,84^ might imply that N57 is irrelevant for the FG phase interactions of pentamer-only spheres. However, Fig. 1g,h documents the opposite: The N57A mutation impeded FG phase entry not only of the hexamer-dominated CLPs and 40-nm spheres but also of the pentamer-only spheres, to the extent that only a faint signal on the FG phase surface remained. Therefore, not only do pentamers’ N57 pockets bind FG peptides but their N57 side-chain amide also engages in energetically relevant hydrogen bonds. The pentamer-only capsid, thus, behaves, by all criteria, like a transport receptor with a general FG-binding capability. The reported binding defect of pentamers^83,84^ is, therefore, selective for the sterically constrained CPSF6 FG peptide, as discussed below (Fig. 7).

### FG phase surface-arrest phenotype

To immerse into the FG phase, the capsid must locally resolve cohesive interactions among FG repeats. This ‘melting’ is associated with a *ΔG* penalty^41^. Full entry into the FG phase, therefore, requires that FG–capsid interactions release more free energy than this penalty. If less free energy is released, capsids will remain at the phase surface, where ‘free’ FG motifs are directly exposed to the aqueous phase.

**Extended Data Fig. 3:**
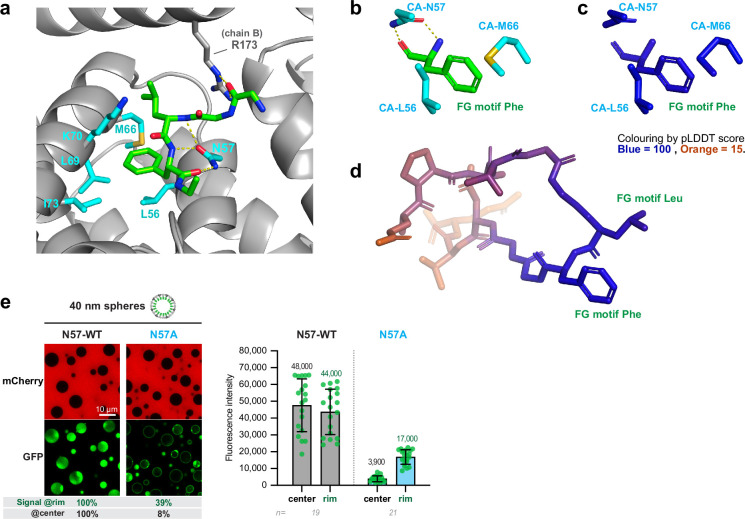
Alphafold 3-modeling confirms that the N57 pocket binds diverse FG repeats in a similar hydrogen bond-stabilized manner. **a**, A mature full-length CA-hexamer was modeled with GLFG peptides bound (the same repeats as in the FG phase assays of Figs. 1, 3–4). Images show just the two CA chains (chain A and chain B) that accommodate the corresponding peptide. The N57 sidechain amide forms two hydrogen-bonds to the backbone of the FG phenylalanine – virtually identically to the FG motif of the CPSF6 peptide (PDB 4B4N, ref. ^66^). The N57 sidechain oxygen forms a third H-bond to the backbone nitrogen of the preceding leucine. Additional H-bonds are formed between the guanidine group of R173 (chain B) and the backbone carbonyl oxygen of the glycine or serine that precedes the LFG motif. The sidechain of the FG phenylalanine engages in extensive hydrophobic contact with L56, M66, L69, (the aliphatic part of) K70, and I73 of chain A. CA-hexamer modeling with SLFG and FSFG peptides are shown in Supplementary Figure S1. **b**, The same highest-ranking structure of a GLFG peptide bound as in (**a**), but panel shows just the phenylalanine of the FG motif, which mediates the key interactions, namely its backbone H-bonding to the sidechain of N57 and the phenyl moiety engaging in hydrophobic contacts with M66 and L56. Coloring is by chain and elements. **c**, Same view as **b** but colored by the local pLDDT score, using the ‘spectrum b’ function of Pymol with indicated settings. The very high score (blue color) indicates a very high confidence of this part of the model (pLDDT= 94). **d**, The entire FG peptide in pLDDT coloring; CA residues are omitted. Note that the pLDDT score deteriorates with sequence distance from the FG motif, reflecting flexibility in accommodating diverse FG repeats. **e**, Wildtype and N57A mutant 40 nm spheres were allowed to partition into GLFG phase, essentially as described in Fig. 1 and ref. ^43^. Note that the repeat units are identical to the peptide modelled in a-d. The N57A mutations caused clear partitioning defects, documenting that the N57 hydrogen bonds contribute to relevant FG interactions. Numbers are means; bars indicate mean ± s.d.; *n* = number of quantified FG particles. Experiments were independently replicated three times, yielding consistent outcomes. Source data

N57A mutant capsids show this ‘surface arrest’ phenotype with all FG phases tested (Fig. 1g,h, Extended Data Fig. 3e and Supplementary Fig. 1), indicating a thermodynamically relevant FG interaction defect and illustrating the relevance of the two N57-mediated hydrogen bonds for locking the peptide backbone of FG peptides. These hydrogen bonds are evident in experimental structures with CPSF6 and Nup153 FG peptides^67^, as well as in AlphaFold models of CA capsomers with captured GLFG, SLFG or FSFG peptides (Extended Data Fig. 3 and Supplementary Fig. 1).

### The highly exposed capsid surface has a very unusual amino acid composition

The residual surface binding of the N57 mutant capsid indicates, however, that the mutant has not lost all FG interactions. As this suggested that other capsid features also contribute, we considered a second (entirely different) binding mode: collective interactions between the surface of a client and the FG phase. The underlying concept^8,29,42^ considers (1) that the FG phase is a solvent for its clients; (2) that any sufficiently exposed residue on the client surface comes into contact with FG repeats when dissolved in the phase (with ~400 mg ml^−1^ FG mass^41^); and (3) that these contacts can be energetically favorable, neutral or unfavorable compared to contacts with water^15,92^.

**Fig. 2:**
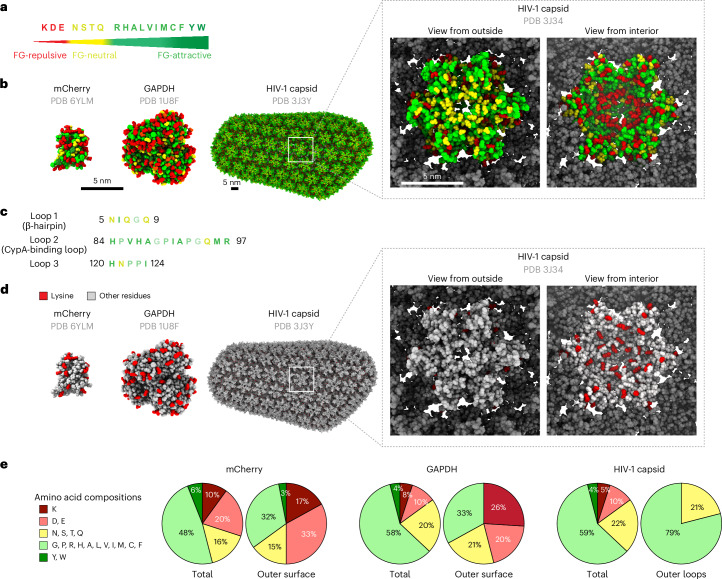
Surface amino acid composition of the HIV-1 capsid. **a**, A surface amino acid scale for partitioning into an FG phase^15^. FG-repulsive residues are colored in red, FG-neutral residues are colored in yellow and FG-attractive residues are colored in green or dark green. **b**, Comparison of the mCherry, GAPDH and HIV-1 capsid surfaces with residues colored according to the FG phase-partitioning scale. Note that FG-repulsive residues (shown in red) are very abundant on the surface of mCherry and GAPDH (an example of cytoplasmic mass protein) and on the inner surface of the capsid but they are absent from protruding parts of the outer capsid surface. There are only a few on the remaining outer surface and these are not well accessible but mostly salt-bridged to FG-attractive arginine and histidine (E113–R97; E98–H84/H87). Scale bars, 5 nm. **c**, Amino acids on the exposed loops of HIV-1 capsid are either FG-attractive or FG-neutral residues. **d**, Comparison of lysines exposed on the surfaces of mCherry, GAPDH and HIV-1 capsid. **e**, Diagrams compare total and surface amino acid compositions, classified by the scale of **a**. Source data

On the basis of this concept, we previously engineered GFP to pass NPCs either very slowly or very rapidly^15^. Variants showed a near-perfect correlation between passage rates and FG phase partitioning. Extreme variants differed 15,000-fold in rate. This engineering exercise also uncovered an amino acid scale for FG interactions (Fig. 2a): negative charges (aspartic acid and glutamic acid) and lysine are strongly FG-repulsive residues, whereas exposed hydrophobic residues, cysteine, methionine, histidine and arginine attract GFP (or other clients) into the FG phase.

The FG-repulsive residues aspartic acid, glutamic acid and lysine are abundantly exposed on the surface of soluble globular proteins (Fig. 2). They provide topological information for the folding process and keep these globular proteins water soluble. They frequently occur in solvent-exposed parts of α-helices, β-sheets and loops. On the protruding part of the outer surface of the HIV-1 capsid, however, they are extremely depleted (Fig. 2b–e). In fact, loop 1 (the β-hairpin), loop 2 (the CypA-binding loop) and loop 3 do not contain a single aspartic acid, glutamic acid or lysine (Fig. 2c,e). This compositional bias is so striking that we suspected a connection to the NTR-like behavior of the capsid.

### The lack of FG-repulsive residues on the capsid surface is key to FG phase entry

**Extended Data Fig. 4:**
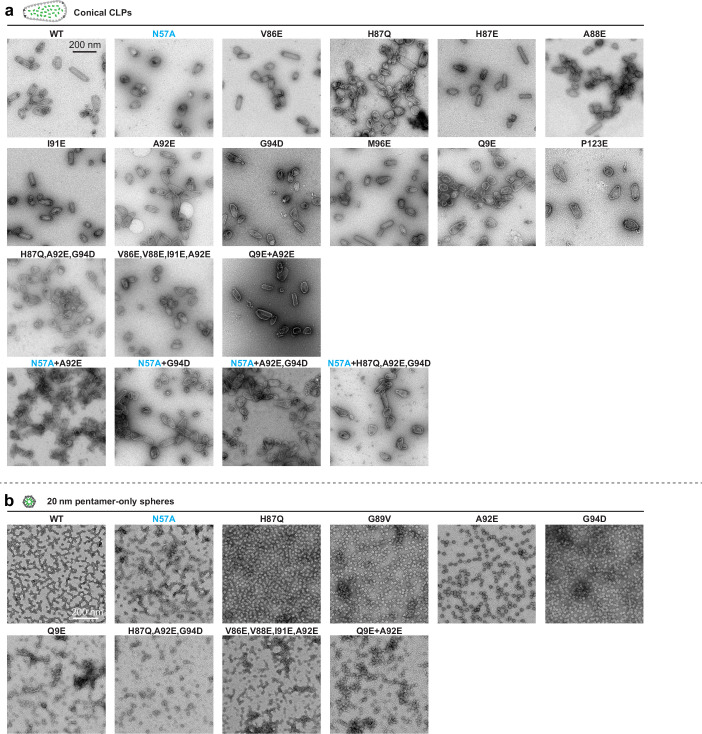
Negative stain electron micrographs of mutant HIV-1 capsids. **a**, Wildtype or mutant CA was recombinantly expressed, purified, and allowed to assemble into cone-shaped CLPs (in the presence of IP6; see Methods). The assemblies were applied to a Superose 6 size exclusion column, where CLPs elute close to the void volume. This can be considered as a first criterion for a faithful CLP assembly. The corresponding fractions were pooled and further analyzed by negative stain electron microscopy. Representative images are shown in this panel and confirm the proper assembly of all mutants for which we describe a transport phenotype. Several other mutants showed defects in capsid assembly (for example, Q7E, M10E/K, V11E, P122E, I124E). These were excluded from the analysis. Experiments were independently replicated three times, yielding consistent outcomes. **b**, Wildtype and mutant pentamer-only spheres were assembled as analyzed in **a**, the differences being (1) that the CA protein contained in addition the G60A+G61P double mutation, which is required to assemble pentamer-only spheres^83^, (2) that the assemblies were smaller than the CLPs (20 nm instead of 100-200 nm), and (3) that these smaller assemblies also eluted later from the Superose 6 column. Scale bars, 200 nm. Experiments were independently replicated two times with consistent outcomes.

To explore this possible connection experimentally, we analyzed 13 additional capsid mutants that were previously reported^64,93–96^. In brief, we assembled CLPs from the respective mutant CA proteins, validated capsid assembly by size-exclusion chromatography and negative-stain EM (Extended Data Fig. 4) and probed the Nup98 GLFG phase entry of these CLPs (which were filled with sinGFP4a to act as a tracer for intact capsids).

**Fig. 3:**
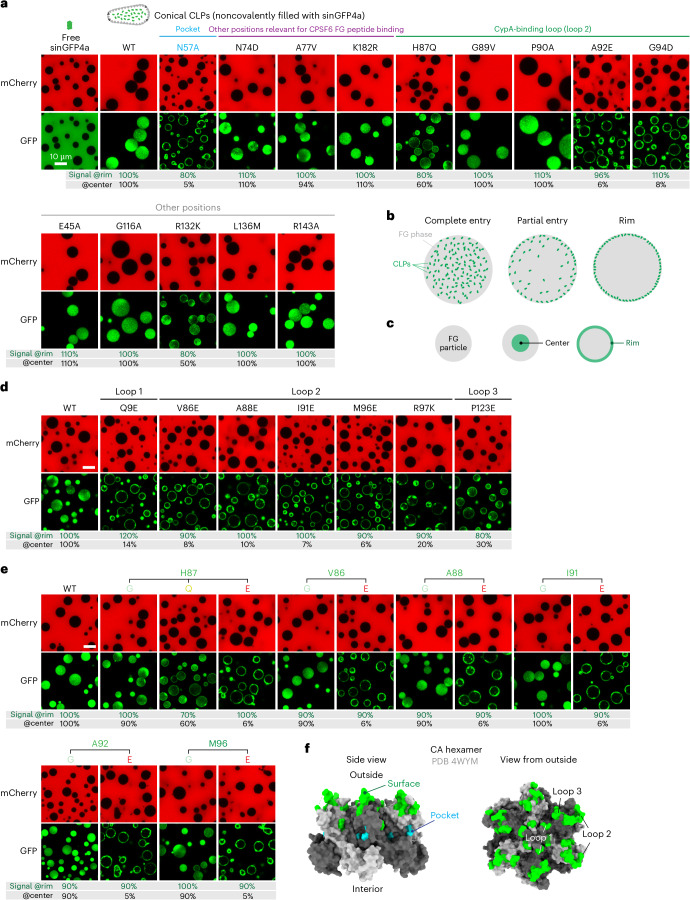
Identification of capsid mutations that impede FG phase partitioning. Partitioning experiments into the FG phase (GLFG 12-mer repeats) were performed as in Fig. 1c but using indicated (wild-type or mutant) CLPs noncovalently filled with sinGFP4a. Experiments were independently replicated three times, yielding consistent outcomes. Scale bars, 10 μm. Negative-stain electron micrographs, validating proper capsid assembly, are shown in Extended Data Fig. 4. **a**, Comparison of wild-type CLPs and CLPs carrying previously reported mutations. Note the strong FG phase-partitioning defect of the N57A, A92E and G94D mutant CLPs. **b**, Illustration of the observed FG phase-partitioning phenotypes. **c**, Illustration of the quantification strategy. Fluorescence signals for the capsid species were integrated separately at the rim and in the center of FG particles, normalized to the wild-type values, and listed for each mutant in **a**,**d**,**e** (more detailed quantifications in Extended Data Fig. 5). **d**, Drastic FG-partitioning defects in rationally designed capsid mutants, where FG-attractive residues were exchanged for FG-repulsive ones (glutamic acid or lysine). **e**, Selected CA positions were mutated to G, Q or E, as indicated. Drastic FG phase-partitioning defects were evident only when an FG-repulsive glutamic acid was introduced. Mutations to an FG-neutral glycine or glutamine had mild effects at best. **f**, A CA hexamer (PDB 4WYM) viewed from the side and the capsid’s outside. Surface mutant positions with FG-partitioning defects are colored in green and the N57 FG-binding pocket is colored in cyan.

**Extended Data Fig. 5:**
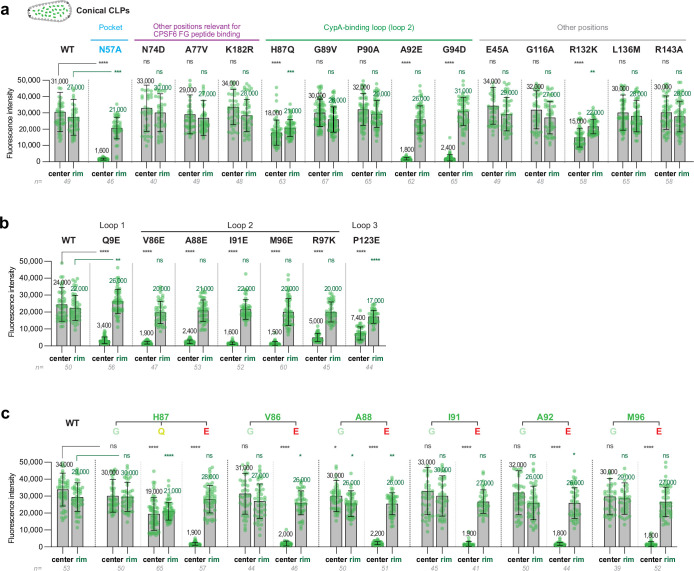
Identification of capsid mutations that caused defects in FG phase-partitioning. The panels a-c provide more detailed quantifications for the partitioning of capsid mutants into the GLFG phase of Fig. 3a-c separately. They are based on larger datasets. Capsid signals at the FG phase surface and in the FG phase center were integrated separately. Numbers are means; bars indicate mean ± s.d.; *n* = number of quantified FG particles. Statistical significance between each mutant and wild-type was determined using an unpaired Student’s *t*-test: *P < 0.05, **P < 0.01, *** *P* < 0.001, **** *P* < 0.0001, ns: not significant (*P* ≥ 0.05). See Supplementary Excel file for tabular data, including *P* values for statistical significance of group differences. Source data

Most mutants entered the FG phase as efficiently as wild-type CLPs (Fig. 3a and Extended Data Fig. 5a). No decrease in partitioning was observed when poorly exposed residues (other than N57) were mutated or when an exposed residue was changed to an equally or more hydrophobic one, such as the G89V and P90A loop 2 mutations, which were previously designed to abrogate interactions with cyclophilin A or the CypH domain^96^. The same was true for the E45A, G116A, L136M or R143A mutations.

The H87Q and R132K mutations caused a ~50% reduction in partitioning. The A92E and G94D mutations in the highly exposed loop 2, however, showed a partition defect similarly strong to the N57A exchange. They arrested the capsids at the FG surface and reduced the partitioning to ~5% of the wild-type level (Fig. 3a–c). This is consistent with the fact that the negatively charged aspartic and glutamic acid residues belong to the most FG-repulsive surface features, which in turn can be explained by the energetically rather unfavorable contacts between negative charges and phenylalanines.

To explore the outer surface of the capsid more systematically, we first identified additional positions that could be mutated without compromising capsid assembly. For this, we again used size-exclusion chromatography and EM to validate faithful capsid assembly (Extended Data Fig. 4). ‘Stable capsid mutants’ were subsequently tested in transport assays (Fig. 3d and see below). A drastic drop in capsid partitioning into the FG phase was evident for six individual mutations to an FG-repulsive glutamic acid (Fig. 3d and Extended Data Fig. 5b). These included the isosteric Q9E exchange at the tip of the β-hairpin of loop 1, the V86E, A88E, I91E and M96E mutations at loop 2 and the P123E exchange at loop 3.

Moreover, the R97K mutation impeded the FG phase partitioning. This preserves a positive charge; however, the two amino acids behave differently toward the FG phase^15^. Lysine is an FG-repulsive residue, whereas arginine is an FG-attractive residue because (1) its planar guanidinium group engages more readily in a cation–*π* interaction with phenylalanines of the FG-repeat domain; (2) it is an excellent hydrogen-bond donor for carbonyl oxygen acceptors in side chains and the polypeptide backbone (discussed in ref. ^41^); and (3) it is more readily transferred from an aqueous to a hydrophobic environment^41,92^. The partitioning defect of the R97K capsid mutant reflects these differences remarkably well.

**Extended Data Fig. 6:**
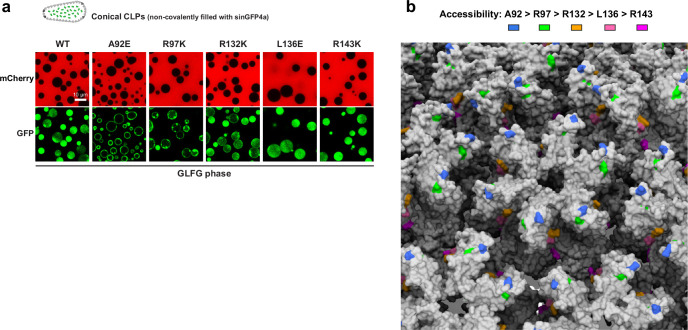
Reduction of FG phase-solubility by FG-repulsive capsid mutations depends on the accessibility of the mutated residues. **a**, The FG phase-partitioning of CLPs was tested as in Fig. 3, using wild-type and indicated mutants. Partitioning was strongly inhibited by FG-repulsive mutations at highly accessible residues (A92E and R97K). When similar mutations were introduced at less accessible positions, partitioning was little affected (R132K) or unaffected (L136E or R143K). Experiments were independently replicated three times with consistent outcomes. **b**, A92, R97, R132, L136 and R143 are shown with indicated coloring in a capsid structure (PDB 3J3Y).

The previously mentioned R132K mutant showed a similar trend but its defect was much weaker, probably because R132 is less exposed (Extended Data Fig. 6). This appears to be a general pattern; R143 is located even deeper in the intercapsomer cavity, which explains why the R143K mutation had no effect on capsid partitioning. Likewise, mutating L136 at the bottom of the intercapsomer cavity to glutamic acid (L136E) had no effect on phase entry, whereas a similar mutation of the highly exposed A92 (A92E) was very detrimental (compared in Extended Data Fig. 6). This nonequivalence emphasizes that the solubility of the HIV capsid in the hydrophobic FG phase is ruled by the surface properties of its protruding and highly exposed parts.

For the FG-partitioning mutants studied here, one could argue that the observed effects are not due to the newly introduced FG-repulsive residues but rather to the loss of the original ones. To address this, we next analyzed different substitutions for the same residue (Fig. 3e and Extended Data Fig. 5c). The mutations of H87 in loop 2 to glycine (H87G) reduced the FG partitioning of the capsid only marginally. The H87Q exchange had a stronger but still mild effect. The H87E mutation, however, was detrimental. It reduced the partitioning ~15-fold and left transport intermediates arrested at the FG phase surface. Thus, the defect cannot be explained by the loss of the imidazole moiety alone. What mattered was indeed the exchange to the FG-repulsive glutamic acid with its negatively charged side chain. The same pattern was observed for mutations of V86, A88, I91, A92, and M96: Exchanges to glycine had only mild effects, whereas mutations to glutamic acid reduced the capsid partitioning >10-fold and led to surface-arrested intermediates.

### Additive effects of capsid-partitioning mutations

**Fig. 4:**
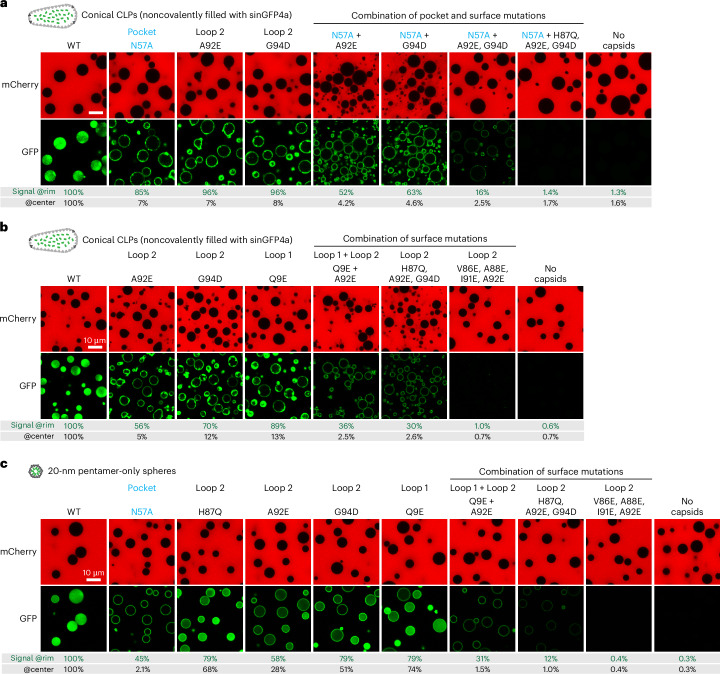
Synergy between ‘loss-of-FG-partitioning’ mutations in hexamer-dominated CLPs and pentamer-only spheres. **a**,**b**, The assay was performed as in Fig. 3, analyzing wild-type CLPs and indicated mutants. Individual mutations caused already strong FG phase-partitioning defects. Combining mutations aggravated the defect up to a complete loss of partitioning. **c**, The assay was performed as in **a**,**b**, the difference being that pentamer-only spheres with a covalent EGFP label were analyzed. These showed a very similar response to the mutations to the CLPs. Experiments were independently replicated three times with consistent outcomes. More detailed quantifications are shown in Extended Data Fig. 7. Scale bars, 10 μm.

**Extended Data Fig. 7:**
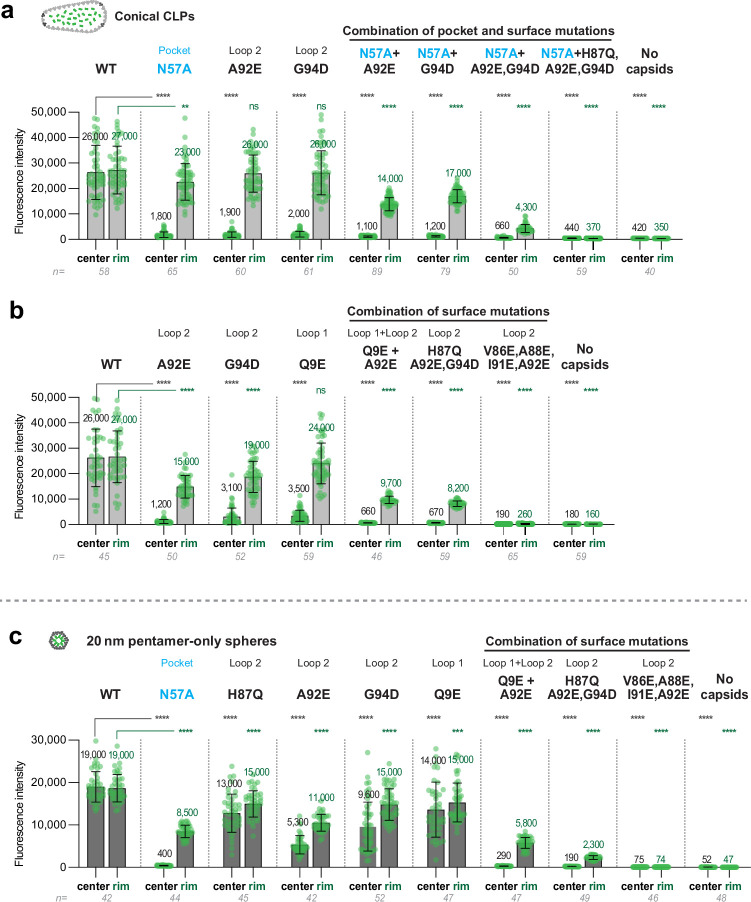
Synergy between surface residues and the FG-binding pocket in FG phase-partitioning of the capsid. The panels a-c provide more detailed quantifications of the partitioning of capsid mutants into the GLFG phase as shown in Fig. 4. Quantification was as in Extended Data Fig. 5. See Supplementary Excel file for tabular data, including *t*-test *P* values. Source data

Up to this point, we identified 12 individual capsid mutants with a clear defect in FG phase partitioning. Yet, all of them retained some FG interactions. A combination of partitioning mutations, however, further reduced the FG phase signal of the capsids up to the point of a complete loss of partitioning and surface binding. This was the case when three FG-repelling surface mutations (H87Q + A92E + G94D) were combined with the N57A pocket mutation (Fig. 4a and Extended Data Fig. 7a).

Likewise, there is also a clear synergy between surface mutations alone, as seen by the greatly reduced signal of the Q9E + A92E double-mutant CLPs (Fig. 4b and Extended Data Fig. 7b). The V86E + A88E + I91E + A92E quadruple-mutant capsid combines four FG-repelling loop 2 mutations and lost all FG phase partitioning and surface binding. This is remarkable because not only does the quadruple mutant keep the N57 pocket intact but loop 2 does not even contact the FG-repeat portion that docks into the N57 pocket—neither in the reported CA hexamer structures with bound CPSF6 or Nup153 FG peptides (PDB 4WYM and 4U0C) nor in structural models with other FG peptides (Extended Data Fig. 3 and Supplementary Fig. 1). Thus, the four negative charges dominate in their repulsion from the condensed FG phase, not only with respect to the still intact N57 pocket but also with respect to all the other FG-attractive residues that still cover the outer surface of the capsid (for example, H87, G94, M96, R97 or P123).

### Capsid mutations that impede FG phase partitioning also interfere with NPC targeting

**Fig. 5:**
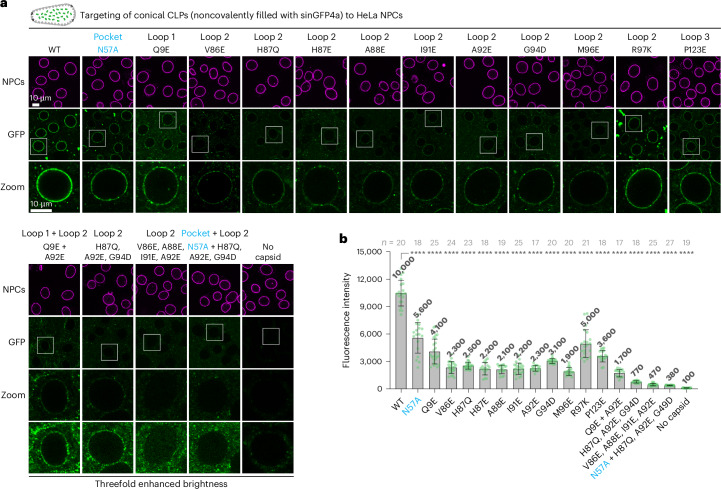
NPC binding requires a lack of FG-repulsive residues on the capsid surface. **a**, Targeting of conical CLPs (noncovalently filled with sinGFP4) to HeLa cell NPCs was as in Fig. 1e. Indicated mutants were tested with identical scan settings as for wild-type capsids. **b**, Quantification of GFP signals on NPCs. Numbers are means; bars indicate the mean ± s.d. (*n* = number of quantified nuclei). Statistical significance between each mutant and the wild type was determined using an unpaired Student’s *t*-test: ****P < 0.001. Tabular data, including *P* values for statistical significance of group differences, are provided in Supplementary Data 1. Scale bar, 10 μm. Experiments were independently replicated three times with consistent outcomes. Source data

In a next step, we compared the targeting of wild-type and mutant CLPs to NPCs of digitonin-semipermeabilized HeLa cells (Fig. 5). To label intact capsids, we again loaded them with sinGFP4a. We not only reproduced the finding that the N57A pocket mutation reduced the NPC targeting^43^, we also observed that all of the FG-repelling surface mutations had an at least equally deleterious effect. In fact, most of these mutations (for example A88E, A92E or M96E) impeded capsid binding to NPCs even more. The effects of these mutations were, again, additive. The V86E + A88E + I91E + A92E and the N57A + H87Q + A92E + G94D quadruple mutants reduced capsid binding to NPCs to background levels.

### Large CLPs completely pass mouse oocyte NPCs when CPSF6 is overexpressed

**Fig. 6:**
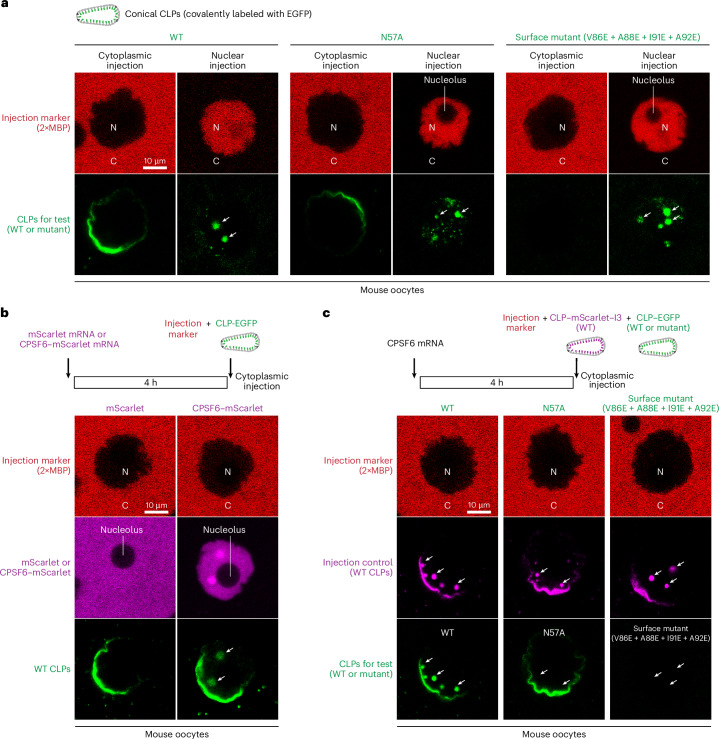
Requirements for NPC passage of CLPs. **a**, Conical CLPs, carrying a C-terminal EGFP label and indicated mutations, were microinjected into fully grown mouse oocytes and imaged 30 min later. The injected compartment, nucleus (N) or cytoplasm (C), was marked by coinjected Alexa647-labeled MBP dimers that do not cross the NE. Wild-type, N57A or V86E + A88E + I91E + A92 quadruple-surface-mutant CLPs accumulated in nuclear speckles after nuclear microinjection. This is a crucial control for the experiment in **c**. None of the large CLPs reached the speckles when microinjected into the cytoplasm. Wild-type and N57A CLPs accumulated at the NE, whereas the quadruple surface mutant did not, consistent with its failure to partition into an FG phase (Fig. 4b). The uneven accumulation of cytoplasmically injected capsids at the NE is because of the essentially irreversible binding upon first encounter of NPCs, combined with slow diffusion of the very large capsids and the resulting concentration gradient in the cytoplasm. **b**, mScarlet or a CPSF6–mScarlet fusion were overexpressed from mRNA that was microinjected into mouse oocytes. After 4 h, EGFP-labeled wild-type CLPs were microinjected into the cytoplasm and imaged 30 min later. The CPSF6 fusion allowed the CLPs to pass NPCs and to accumulate in speckles, where they colocalized with CPSF6. **c**, Unlabeled CPSF6 was overexpressed from microinjected mRNA for 4 h. Then, mScarlet–I3-labeled wild-type CLPs were coinjected with indicated EGFP-labeled mutant CLPs into the cytoplasm and imaged 30 min later. The mScarlet–I3-labeled wild-type CLPs bound NPCs, crossed the NE and accumulated in speckles (indicated by white arrows). EGFP-labeled wild-type CLPs behaved the same. N57A mutant CLPs bound to the NE but failed to pass. Quadruple-surface-mutant CLPs failed to bind NPCs at the NE and failed to accumulate in speckles, again consistent with their failure to partition into an FG phase. Scale bars, 10 μm. Experiments were independently replicated three times with consistent outcomes.

We previously microinjected 40-nm capsid spheres into the cytoplasm of mouse oocytes^43^. These spheres passed NPCs and accumulated at intranuclear structures that probably represent nuclear speckles that are targeted by HIV during genuine infections^57^. When we repeated the experiment with large CLPs (covalently labeled with GFP), we observed only prominent binding to the NE (NPCs) but no intranuclear signal (Fig. 6a). This can be explained by a very slow NPC passage because the same capsids showed prominent speckle binding when injected directly into the nucleus.

We reasoned that capsid release from NPCs into the nucleoplasm was rate limiting and that this slow step could be accelerated by increasing the concentration of the host factor CPSF6 (refs. ^56,64–66,97^). To test this, we microinjected mRNA encoding either mScarlet or a CPSF6–mScarlet fusion. Then, 4 h later, GFP-labeled CLPs were microinjected into the cytoplasm (along with a microinjection marker). Another 30 min later, the oocytes were imaged by confocal laser scanning microscopy (CLSM). This revealed efficient translation of the mRNAs; nonfused mScarlet was evenly distributed throughout nucleus and cytoplasm, sparing only the nucleolus, whereas the mScarlet–CPSF6 fusion was efficiently imported into the nucleus, showing a clean nucleoplasmic signal with a notable speckle enrichment.

**Fig. 7:**
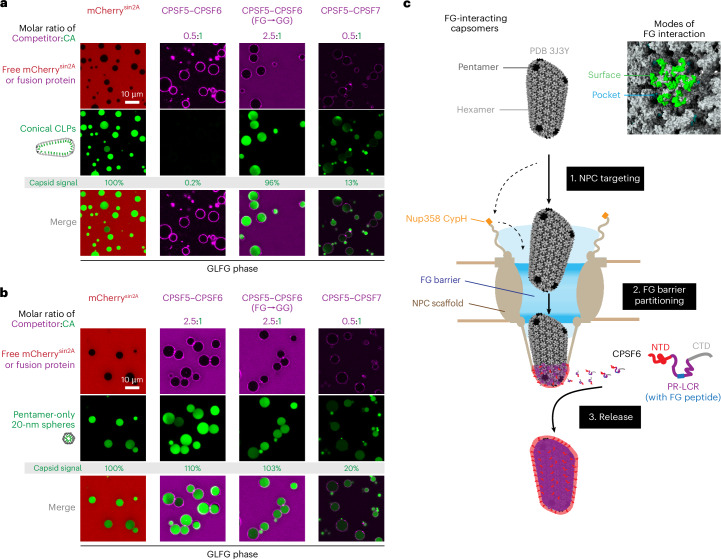
CPSF6 potently antagonizes capsid partitioning into an FG phase. **a**, Capsid partitioning into an FG phase was assayed as in Fig. 3; however, before addition to the GLFG phase, CLPs were premixed with indicated protein complexes. The CPSF5–CPSF6 complex blocked FG partitioning completely. Note also the prominent signal of the CPSF5–CPSF6 complex at the surface of the phase and the lack of capsid colocalization with this signal. This suggests that the CPSF6 complex contains a module that can switch between capsid binding and FG phase partitioning. The CPSF6-F284G mutant complex had no effect on capsid partitioning. CPFS7 (a CPSF6 paralog), which also contains a singular FG motif, competed the partitioning moderately. Molar ratios refer to the tetrameric (A_2_B_2_) CPSF5–CPSF6 or CPSF5–CPSF7 complexes. **b**, Same as in **a**, but FG phase partitioning of 20-nm pentamer-only spheres was analyzed. Note that this capsid species partitioned in a CPSF6-resistant manner. This documents that the rearranged pentamer N57 pocket has no defect in binding ‘normal’ FG peptides but a severe defect in binding the sterically constrained CPSF6 FG peptide. CPSF7 inhibited the partitioning moderately and, thus, binds the pocket in a more tolerant manner than CPSF6. Fluorescence signals for the capsid species were measured in the center of FG particles, normalized to the wild-type values. Experiments were independently replicated three times with consistent outcomes. Scale bars, 10 μm. **c**, Scheme of HIV-1 capsid passage through NPCs. To reach the nucleus, the HIV capsid has to dock to NPCs first. The initial interaction might be a capsid–CypH interaction or a direct targeting to the FG barrier. The capsid then fully partitions into the FG phase, which probably requires disengagement from the CypH domain. CPSF6 extracts the capsid from the NPC barrier and promotes release into the nucleus by using a competing FG peptide and forming a condensate around the capsid, which not only provides a very high local CPSF6 concentration but also masks the FG-attractive surface of the capsid and provides an FG-repulsive outer layer instead. The capsid is, thus, handed over from one phase to another (Supplementary Fig. 3). Source data

Strikingly, when the oocyte nuclei were supplemented with CPSF6, cytoplasmically injected CLPs not only bound to the NE but also passed NPCs and accumulated in nuclear speckles (Fig. 6b). A complementary experiment revealed that the CPSF5–CPSF6 complex is a potent antagonist of capsid partitioning into the FG phase (Fig. 7a). This antagonizing effect was highly specific and completely lost when the singular FG motif of CPSF6 was mutated to a GG motif. Thus, CPSF6 appears to promote the completion of NPC passage by ‘extracting’ the capsid from the FG phase.

### FG-repelling surface mutations block the NPC passage of capsids

To now test capsid mutations for NPC passage defects, we supplemented oocytes with unlabeled CPSF6 before microinjecting GFP-tagged wild-type or mutant CLPs into the cytoplasm. Wild-type CLPs reached the nuclear speckles, whereas the N57A mutant did not (Fig. 6c). The mutant effect was confirmed by two controls. First, coinjected wild-type CLPs, labeled with mScarlet–I3, reached the speckles of the same oocyte. Second, N57A mutant CLPs also accumulated at the speckles when microinjected directly into the nucleus (Fig. 6a). Thus, the N57A mutation causes a genuine NPC passage effect, even though capsid targeting to the NE was still prominent.

The above-characterized V86E + A88E + I91E + A92E surface mutant combines four exchanges of hydrophobic residues for an FG-repulsive glutamic acid. After nuclear microinjection, it effectively bound speckles (Fig. 6a). Following cytoplasmic microinjection, however, it failed to reach intranuclear structures. It did not even show any detectable binding to the NE, indicating a very tight block in NPC targeting and NPC passage. This was obviously a consequence of the failure of these mutant capsids to partition into the FG phase (Figs. 4 and 5). Thus, the presence of FG-attractive residues and the striking absence of FG-repulsive residues in the capsid’s β-hairpin and its two exposed loops are key determinants for both, entering the permeability barrier of NPCs and passing through the pores completely.

## Discussion

The HIV-1 capsid has evolved into an NTR that readily partitions into the FG phase-based permeability barrier of NPCs. It serves as a cargo container for the encapsulated genetic material, shielding it from antiviral sensors in the cytoplasm and delivering it through an intact NE into the nucleus, thus allowing the infection of nondividing cells.

### Both hexameric and pentameric capsomers contribute to FG phase partitioning

**Extended Data Fig. 8:**
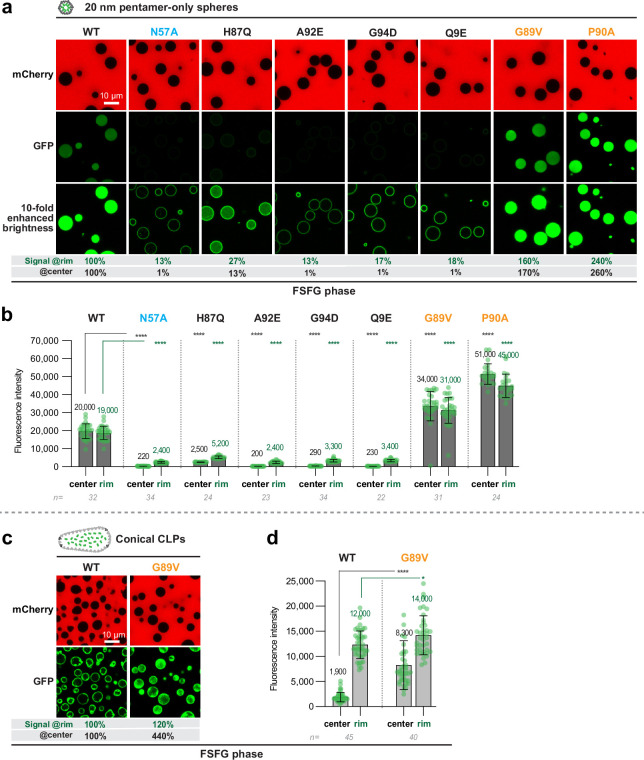
Mutations that block or enhance the capsid-partitioning into an FSFG phase. **a, b**, Partitioning of pentamer-only spheres into the FSFG phase was as in Extended Data Fig. 1, but the indicated CA mutants were also analyzed. The resulting partitioning defects by N57A, A92E, H87Q, G94D, and Q9E exchanges were considerably more severe as compared to the GLFG phase, showing an up to 100-fold reduction as compared to WT. This can be explained by the stronger cohesion of the more hydrophobic FSFG motifs, which necessitates stronger capsid-FG interactions for melting into the phase. Interestingly, the experiment also revealed that the G89V and P90A mutations enhance the partitioning of this capsid species. The effects can be explained by G89V making the surface more hydrophobic and by the P90A exchange allowing for an additional hydrogen-bonding between the nitrogen of the alanine amide and the FG repeats. The P90A enhancement is specific for the pentamer-only spheres. The enhancing effects were not obvious in the GLFG phase experiments, because there, the partitioning was already complete without additional mutations. **c, d**, Partitioning of cone-shaped CLPs into the FSFG phase. The stronger cohesion of this phase results in partitioning defect of wildtype CLPs, which arrest at the surface of the phase. This defect can be compensated by the G89V mutation, which allows for stronger hydrophobic capsid-FG interactions. Quantifications were as in Extended Data Fig. 5. See Supplementary Excel file for tabular data, including *t*-test *P* values. Scale bars, 10 μm. Source data

The capsid is very large, at the very limit of what can possibly pass through an NPC scaffold, and is typically made up of 200–250 hexameric and 12 pentameric capsomers^82,98–102^. It was previously thought that only the hexamers engage in FG interactions^83,84^. However, we have now found that pentamers (represented by pentamer-only T1 spheres^83^) mediate a very efficient FG phase entry as well (Fig. 1). Indeed, CA hexamers and pentamers appear very similar in their FG interactions: efficient NPC targeting and FG phase partitioning of either species require assembly into a capsid structure (Fig. 1c–f). Furthermore, point mutations that impede the FG partitioning of hexamer-dominated capsids also compromise the partitioning of the pentamer-only T1 capsid. This applies to the N57A mutation and the surface loop mutations (Figs. 1g and 4c and Extended Data Figs. 7c and 8a,b).

Although low in count, the contribution of pentamers is probably quite relevant because they are concentrated at the capsid’s narrow end, which appears to insert first^61^ into NPCs and should, therefore, have a particularly high FG-partitioning propensity.

The condensation of FG-repeat domains into an FG phase is driven by cohesive interactions that are of a hydrophobic nature^16,29,41,42,103^. Partitioning of a client into this phase requires a local ‘melting’ of these cohesive interactions. This easily happens when the client (for example, an NTR) binds to (the hydrophobic) FG motifs. Indeed, NTRs belong to the most hydrophobic soluble proteins^29^. This poses a fascinating design challenge: how to make a protein surface ‘NTR-like hydrophobic’ without causing aggregation and noncognate interactions.

### FG-binding pockets and FG wettable surface

Binding pockets for FG motifs are one solution. They have been identified by X-ray crystallography and cryo-EM in several cellular NTRs, such as importin-β, Xpo1/CRM1 or the Mex67–Mtr2 dimer^36,38,40,104,105^. FG-binding pockets can confer excellent binding specificity without fully exposing their hydrophobicity. They contain mostly hydrophobic but also polar residues, whose aliphatic moieties then engage in hydrophobic interactions with FG motifs, while their polar headgroups make hydrogen bonds, for example, with the FG-repeat backbone^36,38,105^. FG-binding pockets appear to confer rather strong FG interactions that can overcome the FG-repelling effects of the typically strong negative charge of cellular NTRs (here, the negative charge is also required to transport highly positively charged cargoes such as histones). It seems reasonable to assume that these details of FG interactions have evolved to optimize cargo transport.

**Extended Data Fig. 9:**
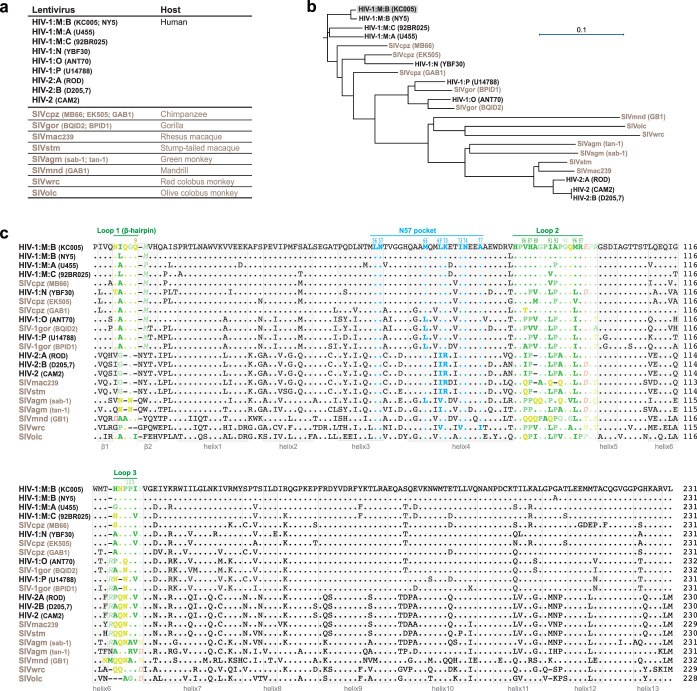
Sequence alignment and phylogenetic analysis of representative lentiviral CA proteins. **a**, Representative lentiviruses and their hosts. **b**, Neighbor-joining phylogenetic tree of lentiviral CA proteins. **c**, A structural alignment of lentiviral CA sequences. HIV-1 isolate KC005 (M group, subtype B) is the CA species analyzed in this study, therefore serving as a reference. Residues of the N57 pocket that contact the FG motif are colored in cyan. Loop1-3 residues are colored according to the amino acid scale of Fig. 2a. Highly exposed residues on the outer loops are in bold. Residues that are not well exposed for FG interactions (either due to being engaged in H-bonds or salt bridges or due to orientation) are in italic. Secondary structures are indicated by light gray boxes. Sequence identifiers are listed in Supplementary Table S7. For sequence variations within the HIV-1: M group, see also refs. ^117,118^ and Table 1.

The HIV-1 capsid has an N57 pocket that occurs in very high multiplicity (given the capsid’s repetitive architecture). It is located between adjacent CA monomers and is much deeper than FG-binding pockets in cellular NTRs. It uses the side chains of L56, M66, L69 and I73 and the aliphatic portion of K70 to form hydrophobic contacts with the FG motif phenylalanine. The side-chain amide of N57 engages in a double or triple hydrogen bond with the backbone of the inserted FG repeat^67^ (Extended Data Fig. 3 and Supplementary Fig. 1). N57 and L56 are absolutely conserved amongst HIV-1, HIV-2 and simian immunodeficiency virus (SIV) (Extended Data Fig. 9). K70, M66, L69 and I73 are also conserved but show some conservative exchanges (K-R, M-L, L-I/V and I-V) (Extended Data Fig. 9). This suggests an ancestral function of this pocket and possibly a common NPC passage mechanism of lentiviral capsids, as discussed below.

However, this pocket is only one part of the story. The entire accessible outer surface of the mature capsid appears to be optimized for ‘wetting’ by the FG phase (Figs. 1–4). It has a highly unusual amino acid composition that follows a previously identified scale for FG phase entry^15^. The key feature is the complete absence of the FG-repulsive residues aspartic acid, glutamic acid and lysine in the exposed loops. This represents a so far unknown mechanism for encoding topogenic information. It is relevant because the introduction of repulsive glutamic acid impedes FG phase partitioning, NPC targeting and NPC passage (Figs. 3–6), completely blocking these steps when mutations are combined. We documented these phenotypes at nine positions: Q9 (loop1), V86, A88, H87, I91, A92, G94 and M96 (loop2) and P123 (loop 3). A similar phenotype was observed for the R97K exchange, where an FG-attractive arginine was replaced by an FG-repulsive lysine. None of these mutations have been observed in isolates from persons with HIV-1 (Table 1), suggesting that they are detrimental to viral fitness. Nevertheless, we assume that they still allow infection of proliferating cells, in which a capsid passage through NPCs is not a strict requirement.

**Table 1:** Circulating HIV strains strictly avoid FG-repelling capsid mutations

Mutation	Position	FG phase partitioning	NPC targeting	Residues in naturally occurring strains
N57A	FG pocket	Severe defect	Reduced	N
V86E	Loop 2	Severe defect	Reduced	V, A, P, I, Q, T, M, L
H87E	Loop 2	Severe defect	Reduced	H, Q, P
A88E	Loop 2	Severe defect	Reduced	A, V
I91E	Loop 2	Severe defect	Reduced	I, V, N, A, F, H, L, P, Y, T, Q, M
A92E	Loop 2	Severe defect	Reduced	A, P, Q, V
G94D	Loop 2	Severe defect	Reduced	G, N
M96E	Loop 2	Severe defect	Reduced	M, I, L, V
R97K	Loop 2	Reduced	Reduced	R
Q9E	Loop 1 (β-hairpin)	Severe defect	Reduced	Q, H, A
P123E	Loop 3	Reduced	Reduced	P, A, S, V, G

The table correlates capsid mutations that interfere with FG phase partitioning with substitutions at the same positions found in isolates from persons with HIV-1 M subgroup strains (shown in order of frequency). Note the absence of FG-repulsive substitutions (to lysine, glutamic acid or aspartic acid) in naturally occurring strains, indicating that such mutations compromise replication fitness. N57A compromises FG phase partitioning and the interaction with CPSF6. Sequence data were taken from previous studies^117,118^. The amino acid scale is shown in Fig. 2a, conservation among lentiviruses is shown in Extended Data Fig. 9, data for FG partitioning are shown in Figs. 3 and 4 and Extended Data Fig. 8 and data for NPC targeting are shown in Fig. 5.

One would expect enhanced FG phase partitioning if capsid surface residues were changed to more FG-attractive ones. Such an effect is not obvious with our standard GLFG phase that already fully absorbs the wild-type CLPs. However, such an enhancement is evident for a stricter FSFG phase, which does not allow full entry of the wild-type CLPs. Here, the G89V mutant capsid enters four times more effectively (Extended Data Fig. 8c,d).

**Extended Data Fig. 10:**
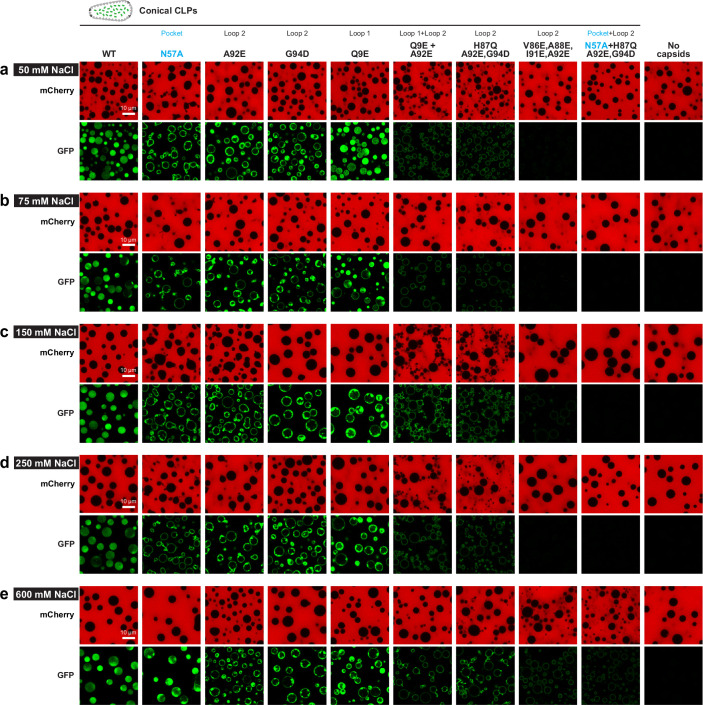
FG pocket and capsid surface residues contribute different qualities of FG interactions. Partitioning experiments into the GLFG 12mer FG phase of CLP mutations were performed as in Fig. 3, but the NaCl concentration was varied from 50 mM to 600 mM as indicated in panels a-e. Note that wildtype CLPs showed a perfect FG phase partitioning at all salt concentrations. N57A mutant CLPs showed a strong partitioning defect at 50 mM, 75 mM, 150 mM, and 250 mM NaCl; however, at 600 mM NaCl, the N57A mutant CLPs efficiently entered the FG phase. The A92E and G94D mutations showed a strong partitioning defect at any salt concentration. The Q9E mutant CLPs entered the phase at low salt, but showed a partitioning defect at higher salt. These different behaviors indicate that N57 and the critical surface residues mediate interactions of different qualities. Experiments were independently replicated two times with consistent outcomes.

N57 engages in a polar hydrogen-bond interaction with the FG-repeat backbone, whereas the capsid surface residues analyzed here probably contribute mostly hydrophobic contacts. The different qualities of these interactions become experimentally apparent when the salt concentration is varied: wild-type CLPs enter the GLFG phase at low (50 mM NaCl), medium (150–250 mM) or high (600 mM) salt concentrations (Extended Data Fig. 10). The N57A mutant fails to partition at low and medium salt concentrations but enters the phase at 600 mM NaCl, probably because this strengthens the remaining hydrophobic capsid surface–FG interactions. X-E surface mutations, however, could not be rescued by high salt. In fact, the Q9E mutant even shows an inverse salt effect.

### A hydrophobic yet aggregation-resistant capsid surface

The three outer loops are dominated by FG-attractive residues. The most attractive ones, tryptophan, tyrosine and phenylalanine, however, are suspiciously missing (Fig. 2 and Table 1). This is perhaps because highly exposed tryptophans or tyrosines are very prone to promiscuous interactions. We observed this in our previous engineering study on transforming GFP into an NTR^15^, where placing additional tryptophans or tyrosines onto the GFP surface greatly enhanced FG phase partitioning and the rate of NPC passage but also conferred massive aggregation with nuclear and cytoplasmic structures.

By rational design, followed by mutagenesis and screening of many variants, we eventually obtained rapidly translocating NTR-like GFP variants highly specific for NPCs and the FG phase^15^; however, strikingly, they all lacked fully exposed tyrosines and tryptophans. It is remarkable that the evolution of the capsid into a specific NTR arrived at a similar solution.

This must have occurred under strong selective pressure, as aggregation propensity scales with size and thousands of cytoplasmic and nuclear proteins were potential aggregation partners. There are fascinating parallels here to an antibody response by the immune system, which not only aims for strong target binding but also selects against broad cross-reactivity with myriads of undesired (self-)targets. CDRs (complementarity-determining regions) of early-stage antibodies often contain exposed tyrosines, perhaps because tyrosine-containing CDRs have such a high propensity to engage in interactions^106^. In well-affinity-matured antibodies, however, tyrosines are typically replaced by more sophisticated and target-specific hydrophobic arrays^107^. In this sense, we consider the HIV-1 capsid to be a highly selectivity-matured entity—a masterpiece of evolution, created through very deep sampling of sequence space and the ‘testing’ of extremely large numbers of variants during infection.

### Evolutionary pressure against surface lysines

To pass NPCs and thereby provide the selective advantage of infecting nondividing cells, the capsid had to be highly optimized. Could this have evolved in a single step? Or did some other evolutionary pressure pave the way first? Possibly, at least as far as the suspicious absence of accessible lysines is concerned. Lysines are not only FG-repulsive residues but also acceptors for ubiquitin conjugation. We now propose that their elimination from the accessible outer surface (Fig. 2d) came first, to protect incoming capsids from proteasomal degradation. Indeed, even the CA N terminus is refractory to ubiquitin modification, as it is a secondary amine (proline) and is buried in the mature capsid^99,100,102^.

### FG-attractive capsid features are more ancient than HIV

The N57 FG-binding pocket is well conserved amongst lentiviruses, as are the outer capsid loops, which have a similar amino acid composition to HIV-1 (see above, Table 1, and Extended Data Fig. 9). This suggests that lentiviral capsids share the ability to partition into the FG phase and pass NPCs, which would explain why lentiviruses are generally able to infect nondividing cells. Indeed, our preliminary data indicate that HIV-2 and SIV capsids can also target the NPC autonomously and partition into an FG phase, albeit not quite as efficiently an HIV-1 M group capsid.

In contrast, capsids of simple retroviruses (such as the mouse leukemia virus^108,109^) lack the FG-attractive extension of loop 2. This suggests that they cannot pass NPCs in the same way as HIV, which would explain why they infect quiescent cells only with marginal efficiency^54,110^.

### Escape from the energy well of the FG phase

HIV-1 capsids partition so strongly into the FG phase that the outside signal becomes undetectable^43,44^ (Figs. 1, 3 and 4, and Extended Data Figs. 1, 3, 5–8 and 10). Capsids are, thus, trapped in a deep energy well. How can they escape? During cellular transport, the RanGTPase system releases cargo from importin β-type NTRs and, thus, also from the NPC barrier through nuclear RanGTP binding to importins or cytoplasmic GTP hydrolysis in cargo–exportin–RanGTP complexes^4,88^. The capsid, however, is not a RanGTP effector. HIV, therefore, needs a different strategy to release its genetic material from NPCs. The following strategies come to mind:

1. Destruction of NPCs. Capsids can crack NPCs^111^. Yet, cracks are unlikely to trigger capsid escape from the FG phase because the NPC scaffold elements and, thus, the FG mass stay in place. Furthermore, such cracks do not occur in all infectable cell types. We regard them as ‘collateral damage’ and not as a requirement for capsid passage through NPCs. Anyway, it appears that the nucleocytoplasmic barrier of mouse oocytes did not ‘suffer’ from cracks. It continued to exclude our injection marker even after large numbers of capsids were inserted into and passed through NPCs (Fig. 6).
2. Disassembly of still NPC-trapped capsids, leading to vDNA release. As FG phase partitioning is favored by capsid assembly^43,44^ (Fig. 1 and Extended Data Fig. 1), the inverse should also hold true, namely, capsid stabilization by immersion into the FG phase. However, once reverse transcription has sufficiently increased the volume of the enclosed nucleic acids^112,113^, FG phase-trapped capsids will also burst and release the viral genome into either the nucleus or the cytoplasm. This pathway likely permits infection if capsids (or capsid mutants) fail to interact with CPSF6 (see ref. ^95^ and below).
3. The most plausible mechanism for a release of intact capsids from NPCs is an energy input from nuclear binding events that disengage the Nup FG repeats from the capsid. Such a mechanism would allow for a directional capsid release into the nucleoplasm (Fig. 7). Indeed, CPFS6 is the perfect candidate for terminating the capsid’s NPC passage. It is a host factor for infection, it binds the capsid directly, it participates in the nuclear events during the establishment of infection^64–66,97^ and capsids accumulate at NPCs when CPSF6 is depleted^56^. We have now directly demonstrated that the partitioning of fully assembled capsids into the FG phase is potently antagonized by the CPSF5–CPSF6 complex and that the CPSF6 paralog CPSF7 has a similar albeit weaker effect (Fig. 7a,b).

Following cytoplasmic microinjection into CPSF6-supplemented mouse oocytes, large CLPs inserted into NPCs, completed passage and accumulated at nuclear speckles (Fig. 6). With just endogenous CPSF6 levels, however, they remained arrested at NPCs. This can be explained by a lower CPSF6 availability in late-stage oocytes than in typical HIV target cells. At nonlimiting CPSF6 levels, the capsid passage through NPCs was comparably efficient, considering that oocytes were loaded with far more capsids (~10,000) than during a genuine infection and images were acquired already 30 min after injection, which is fast for a transport experiment in oocytes, where diffusion distances and, thus, diffusion times far exceed those in somatic cells. It is also fast compared to the estimated 1.5-h capsid dwell time at NPCs during a genuine infection^114^. The kinetics of NPC passage and release of these large capsids (Fig. 6), thus, appear to be within a physiologically plausible range.

To ‘extract’ the capsid from its highly multivalent interactions with the FG phase, CPSF6 must compete against an extremely high local FG concentration. Given this challenge, CPSF6’s block of capsid partitioning is remarkably strong—far stronger than that of the N57A mutation that essentially inactivates the FG-binding pocket (Fig. 7). This suggests that CPSF6 not only obstructs the pocket but also masks the FG-attractive surface identified here. For this discussed antagonistic effect, it appears critical that the ‘mask’ is repelled from the FG phase while covering the capsid.

Surface masking probably involves fuzzy hydrophobic interactions with the proline-rich low-complexity region (PR-LCR) that flanks the CPSF6 FG peptide and contributes to capsid binding^115^. The PR-LCR is extremely depleted of charged FG-repulsive residues but enriched in hydrophobic ones including phenylalanines (Supplementary Fig. 3), thereby resembling an Nup98 FG domain. Thus, just as the compositionally biased capsid surface is attracted to the FG phase, it should attract the PR-LCR of CPSF6. This attraction allows capsids to seed CPSF6 condensates^97,116^, which then provide a very high local CPSF6 concentration and, thus, avidity to the interaction.

An intriguing twist is the negatively charged CPSF6 N terminus, which appears to be FG-repulsive and immiscible with the local PR-LCR condensate. This suggests a layered arrangement around the capsid; the PR-LCR condensate forms the inner layer, masks the FG-attractive surface, blocks the FG pocket (through the embedded FG motif) and provides condensate-stabilizing interactions (perhaps together with the C-terminal mixed-charged domain^97^). The negatively charged (FG-repulsive) CPSF6 N terminus is excluded from the condensate and, thus, forms an FG-repulsive outer layer.

This arrangement would, thus, cause the capsid to switch from an FG-attracted to an FG-repulsive species, explaining capsid release from the FG phase of NPCs. The nuclear localization of CPSF6 ensures that this release occurs into the nucleus and makes the capsid transport directional. In this respect, CPSF6 appears to be analogous to RanGTP, which also acts as a directional switch, namely, as a nucleus-specific release factor for cellular cargoes from importins and, thus, from NPCs^4^.

## Methods

### DNA sequences for recombinant protein expression

All recombinant proteins used in this study were produced in *Escherichia coli*, using codon-optimized genes, His_14_–NEDD8 or His_14_–SUMO tags and a purification strategy that includes binding to a Ni^2+^ chelate matrix and proteolytic release by NEDP1 or SenP1/Ulp1 (ref. ^119^). Expression vectors are listed in Supplementary Tables 2–5.

### Assembly and purification of conical CLPs

His_14_–bdSUMO-tagged CA-P1A was expressed in NEB Express *E.* *coli* cells (New England Biolabs, C2523). Here, the P1A mutation is required for tag removal (that is, to allow SUMO cleavage). Induction was performed with 0.1 mM IPTG at 18 °C for 16 h. Cells were harvested by centrifugation, resuspended in lysis buffer (50 mM Tris-HCl pH 8.0, 300 mM NaCl, 20 mM imidazole and 1 mM TCEP) and lysed by a freeze–thaw cycle followed by sonication. The lysates were cleared by ultracentrifugation, the soluble fractions were bound to Ni^2+^ chelate beads for 1 h at 4 °C, beads were washed with wash buffer (50 mM Tris-HCl pH 8.0, 40 mM imidazole, 300 mM NaCl and 1 mM TCEP) and proteins were eluted by tag cleavage with 100 nM bdSENP1 protease in cleavage buffer (50 mM Tris-HCl pH 8.0, 20 mM imidazole, 300 mM NaCl and 0.5 mM TCEP) for 3 h at 4 °C.

The tag-free proteins were concentrated to approximately 20–30 mg ml^−1^. IP6-assisted assembly^74^ into CLPs essentially followed a published protocol^83^ with minor modifications. The buffer of the CA protein was exchanged to 25 mM MES pH 6.0, 50 mM NaCl and 1 mM TECP. Assembly was initiated by adding 0.5 volumes of 75 mM MES pH 6.0, 150 mM NaCl, 15 mM IP6, 3 mM TCEP and shifting the temperature to 37 °C for 2 h. The final volume was 500 µl and the CA concentration was 12 mg ml^−1^. We generated more homogeneous CLPs (size from ~60 × 100 nm to ~80 × 160 nm) with this protocol than with the previous one^43^, which produced larger particles in a different assembly buffer (50 mM Tris pH 8.0, 1 M NaCl and 0.1 mM IP6).

Assembled CLPs were pelleted by centrifugation in a 5424R Eppendorf microcentrifuge (FA-45-24-11 rotor) at 21,000*g* for 10 min and resuspended in gel-filtration buffer (25 mM Tris-HCl pH 8.0, 150 mM NaCl, 0.5 mM IP6 and 0.5 mM TCEP); aggregates were removed by centrifugation at 21,000*g* for 5 min. Note that the pH shift prevents the pelleting of properly assembled CLPs in the second centrifugation step. The CLPs in the supernatant were further purified by size-exclusion chromatography on a Superose6 Increase 10/300 GL column (equilibrated in gel-filtration buffer), where they eluted in the void volume. Assembly was performed either in the presence of 2 mM soluble sinGFP4a (ref. ^15^) as a noncovalently encapsulated tracer or with CA–EGFP or CA–mScarlet–I3 fusion^120^ used as a tracer in a 1:6 molar ratio to the unlabeled CA.

CLPs with the Q9E+A92E, H87Q+A92E+G94D, N57A+A92E+G94D, N57A+H87Q+A92E+G94D and V86E+A88E+I91E+A92E mutations did not pellet in the postassembly centrifugation step, probably because of charge repulsion. They were, therefore, directly applied to the Superose 6 column, after removing aggregates by a 5-min 21,000*g* centrifugation step.

### Assembly and purification of 40-nm capsid spheres

His_14_–bdSUMO-tagged CA-P1A with the additional N21C and A22C mutations^86^ was expressed and purified as described above and concentrated to approximately 6 mg ml^−1^. Assembly was performed by dialysis against 50 mM Tris-HCl pH 8.0, 1 M NaCl and 0.1 mM IP6 for 24–48 h. Assembled CLPs were further purified by size-exclusion chromatography on a Superose6 Increase 10/300 GL column equilibrated in 25 mM Tris-HCl pH 8.0, 500 mM NaCl and 0.5 mM IP6, where they eluted near the void volume.

### Assembly and purification of 20-nm pentamer-only spheres

For assembly of 20-nm pentamer-only spheres, the His_14_–bdSUMO-tagged CA P1A+G60A+G61P mutant was purified as described above and concentrated to approximately 20–30 mg ml^−1^. Assembly into 20-nm capsids followed a published protocol^83^ with minor modifications. Briefly, the buffer of the CA protein was exchanged to 25 mM MES pH 6.0, 50 mM NaCl and 1 mM TECP; assembly was initiated by adding 0.5 volumes of 75 mM MES pH 6.0, 150 mM NaCl, 15 mM IP6 and 3 mM TCEP and allowed to proceed in a volume of 500 µl for 2 h at 37 °C and a CA concentration of 12 mg ml^−1^. Assembled CLPs were further purified by size-exclusion chromatography on a Superose6 Increase 10/300 GL column equilibrated in 25 mM Tris-HCl pH 8.0, 150 mM NaCl, 0.5 mM IP6 and 0.5 mM TCEP.

### Fluorescence labeling

The anti-Nup133 nanobody xhNup133–Nb2t was labeled with Alexa Fluor 647 C_2_ maleimide (Thermo Fisher) through two ectopic cysteines at the N and C termini as described previously^89^, reaching a density of labeling of 2. The maltose-binding protein (MBP) tandem dimer (2×MBP) was also labeled with Alexa Fluor 647 C_2_ maleimide but through a single cysteine at the C terminus.

### Negative-stain EM

Samples were bound to a glow-discharged carbon foil covered 400-mesh copper grid. After successive washes with water, samples were stained with 1% uranyl acetate in water and examined at room temperature on a Talos L120C transmission EM instrument (Thermo Fisher Scientific).

### Digitonin-permeabilized cell assays

HeLa K cells (RRID: CVCL_1922) and XTC-2 cells (RRID: CVCL_5610)^91^ were obtained from the European Cell Culture Collection, authenticated by the manufacturer and tested negative for *Mycoplasma*. HeLa cells were grown at 37 °C in DMEM (high glucose), supplemented with 10% heat-inactivated fetal calf serum (FCS), antibiotics (‘AAS’, Sigma-Aldrich) and 5% CO_2_. XTC-2 cells were cultivated at 22 °C in 70% Leibovitz medium (diluted with water), 10% FCS and AAS antibiotics.

Cells were seeded on eight-well μ-slides (IBIDI) to 70% confluence. Plasma membranes were permeabilized^42,121^ by treating the cells with 30 μg ml^−1^ digitonin (water-soluble fraction) in transport buffer (20 mM HEPES–KOH pH 7.5, 110 mM (HeLa) or 80 mM (XTC-2) potassium acetate, 5 mM magnesium acetate, 0.5 mM EGTA and 250 mM sucrose) for 3 or 6 min at 25 °C (with gentle shaking), followed by three washing steps in transport buffer. Permeabilized cells were then incubated for 30 min with 40 nM Alexa647-labeled xhNup133–Nb2t nanobody and EGFP (3 μM), CA–EGFP (1 μM), conical CLPs, 40-nm capsid spheres of HIV-1 or 20-nm pentamer-only spheres (with CA concentrations of 5, 1 and 2 μM). The samples were then directly scanned with a Leica SP8 CLSM instrument (equipped with a ×63 oil objective and HyD GaAsP detectors), with sequential excitation at 488 and 638 nm.

### mRNA for microinjection

Mouse CPSF6 (Consensus CDS: CCDS78898.1) and mScarlet^122^ cDNAs were cloned into pGEMHE^123^, which contains a T7 promoter, *Xenopus* globin 5′ and 3′ untranslated regions and a poly(A) tail. Plasmids were linearized by AscI (New England Biolabs) before in vitro transcription using the HiScribe T7 ARCA mRNA kit (New England Biolabs, E2060S). mRNAs were purified with the RNeasy mini kit (Qiagen, 74104) and 4 pl of 1 µM mRNA was cytoplasmically injected.

### Microinjections

As previously described^124^, mouse oocytes were obtained from ovaries of 9-week-old CD1 mice that were maintained in a specific-pathogen-free environment according to the Federation of European Laboratory Animal Science Association guidelines and recommendations, in a facility registered with the designated authority (LAVES, reference no. 33.23-42508-066-§11; January 31, 2024) according to §11 (Section 1) of the Animal Welfare Law of the Federal Republic of Germany.

Fully grown oocytes were kept arrested in prophase in homemade M2 medium supplemented with 250 μM dibutyryl cyclic adenosine monophosphate under paraffin oil (NidaCon) at 37 °C. Labeled CLPs, along with the Alexa647-labeled 2×MBP injection marker, were microinjected into cytoplasm or nucleus of oocytes, as previously described^43^. Oocytes were imaged about 30 min after microinjection.

### FG phase assays

The assays were performed as previously described^88^ with minor modifications. In brief, 1 mM FG domain stocks were prepared in 2 M (GLFG repeats) or 4 M guanidinium hydrochloride (all other repeat domains). Phase separation was initiated by rapid dilution of the FG domain stock with 25 volumes (GLFG repeats) or 50 volumes (other repeats) of assay buffer (50 mM Tris-HCl pH 7.5, 150 or 250 mM NaCl and 0.5 mM IP6), followed by a further fourfold dilution in buffer with indicated fluorescent probes. The resulting mixture was pipetted on collagen-coated 18-well μ-slides (IBIDI) and FG particles were allowed to settle on the bottom for 1 h before confocal scans were taken. Salt effects of the assay are detailed in Extended Data Fig. 10.

Partition coefficients were calculated as the integrated raw signal within independent FG particles (in) divided by the signals reference areas in outside regions (out). The background was not subtracted, which means that the numbers of high partition coefficients were still underestimated. Plots are shown for representative FG particles (with 5–10-μm diameters). Images were analyzed in FIJI 2.9.0 and the exported data were further processed in GraphPad Prism 10.4.1

The sequences of FG domains used in FG phase assays are shown in Supplementary Table 1.

### Structure modeling

The Alphafold3 server^125^ was used for all modeling shown in Extended Data Fig. 3a–d and Supplementary Fig. 1a,b. FG binding was modeled with six copies of the wild-type HIV-1:M CA and six copies of the respective FG peptide. The following sequences were used: CPSF6, PVLFPGQPFGQPPLG; GLFG, QPATGGLFGGNTQ; SLFG, QPATGSLFGGNTQ; FSFG, NTQPATGFSFGGNTQPATG; CA, PIVQNLQGQMVHQAISPRTLNAWVKVVEEKAFSPEVIPMFSALSEGATPQDLNTMLNTVGGHQAAMQMLKETINEEAAEWDRLHPVHAGPIAPGQMREPRGSDIAGTTSTLQEQIGWMTHNPPIPVGEIYKRWIILGLNKIVRMYSPTSILDIRQGPKEPFRDYVDRFYKTLRAEQASQEVKNWMTETLLVQNANPDCKTILKALGPGATLEEMMTACQGVGGPGHKARVL.

### Statistics and reproducibility

The s.d. was represented by error bars on the *y* axis for bar graphs plotted from the mean value of the data. Statistical significance based on an unpaired Student’s *t*-test was calculated using the *t*-test function of Mathematica 14.3.0 for MacOS. *P* values are listed in Supplementary Data 1. No statistical method was used to predetermine sample size. No data were excluded from the analyses. The experiments were not randomized and investigators were not blinded to allocation during experiments or outcome assessment. Experiments were independently replicated at least two times.

### Reporting summary

Further information on research design is available in the Nature Portfolio Reporting Summary linked to this article.

## Online content

Any methods, additional references, Nature Portfolio reporting summaries, source data, extended data, supplementary information, acknowledgements, peer review information; details of author contributions and competing interests; and statements of data and code availability are available at 10.1038/s41594-025-01684-5.

## Supplementary information

Supplementary Information Supplementary Figs. 1–3, Tables 1–7 and References.

Reporting Summary

Supplementary Data 1 Tabular data for quantifications.

## Source data

Supplementary Data 2 Statistical source data for Supplementary Fig. 2.

Source Data Figs. 1, 2, 5 and 7, and Extended Data Figs. 1, 3, 5, 7 and 8 Statistical source data.

## Data Availability

All data that support the findings of this study are available within the Article and Supplementary Information. Source data are provided with this paper.
