# Supplementary material for: Nuclear pore passage of the HIV capsid is driven by its unusual surface amino acid composition
Source: Nat Struct Mol Biol. 2025 Oct 9;32(12):2476–91. doi: 10.1038/s41594-025-01684-5 (PMC12700808; doi:10.1038/s41594-025-01684-5)
Supplement: Supplementary file 1 — Supplementary Figs. 1–3, Tables 1–7 and References. [file 41594_2025_1684_MOESM1_ESM.pdf]

nature structural & molecular biology

Supplementary information https://doi.org/10.1038/s41594-025-01684-5

Nuclear pore passage of the HIV capsid is driven by its unusual surface amino acid composition

In the format provided by the authors and unedited

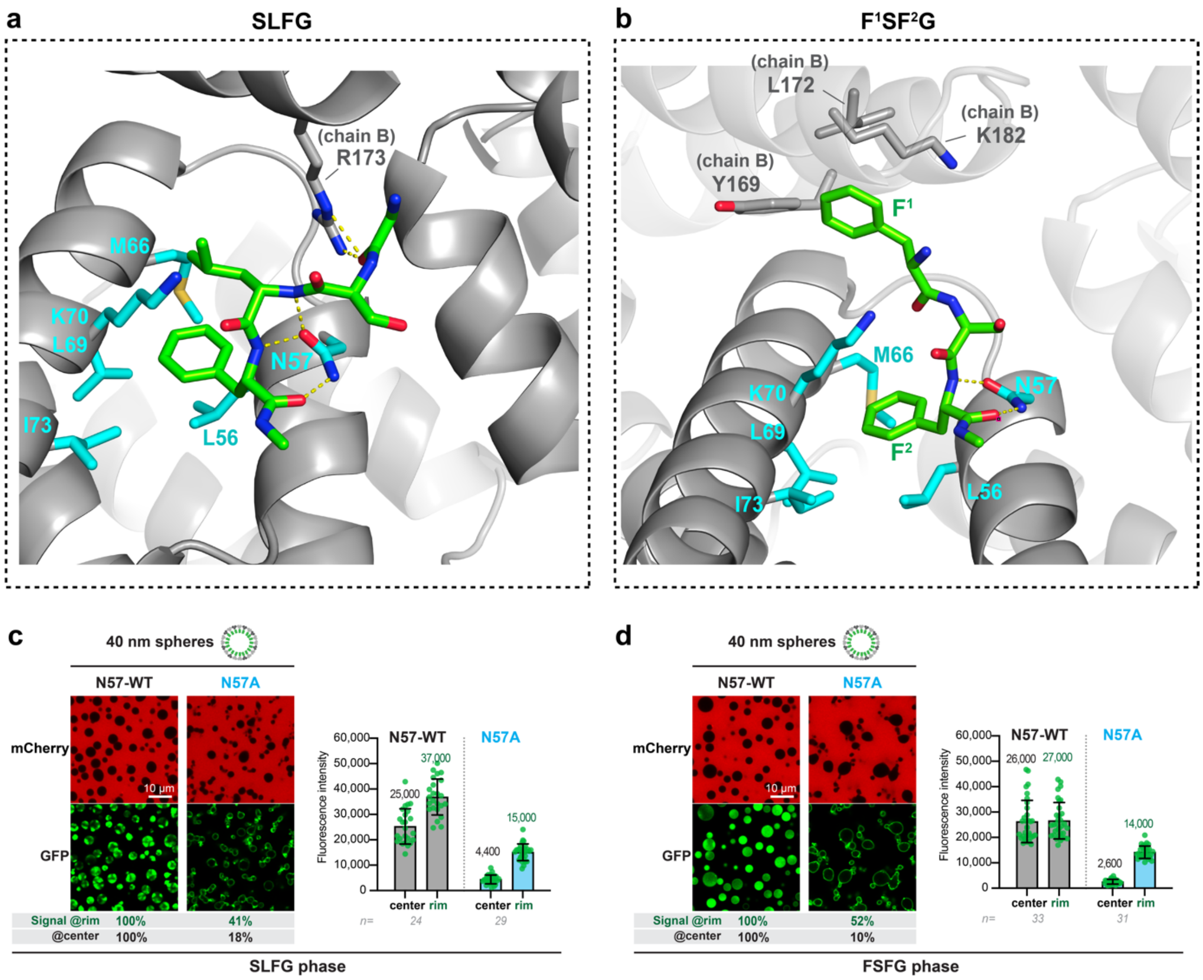

Supplementary Figure S1. Alphafold 3-modeling of SLFG and FSFG peptide binding into the N57 pocket.

a, Modelling of an SLFG peptide docked into the N57 pocket of an hexameric capsomer was as described in Extended Data Fig.3, the only difference being the sequence of peptide (QPATG**SLFG**GNTQ). The hydrophobic contacts of the FG motif phenylalanine sidechain as well as the H-bonding of its backbone to N57 are virtually identical to those of the CPSF6 or GLFG peptide. The non-conserved parts of the peptide differ in their contacts. For clarity, only the SLFG motif is shown.

b, Model of a docked FSFG peptide of the sequence NTQPATG**FSFG**GNTQPATG. Only the FSFG part of the peptide is shown. The FG motif phenylalanine makes again virtually identical contacts to those of the other FG peptides. The preceding phenylalanine (F^1) of the FSFG peptide makes additional hydrophobic contacts with Y169, L172, and (the aliphatic part of) K182 of chain B.

c, d, Wildtype and N57A mutant 40 nm spheres were allowed to partition into SLFG and FSFG phases essentially as described in Extended Data Fig.3e. The N57A mutation reduced capsid-partitioning into the SLFG phase and the FSFG phase, validating the relevance of N57 H-bonds for general FG peptide-binding.

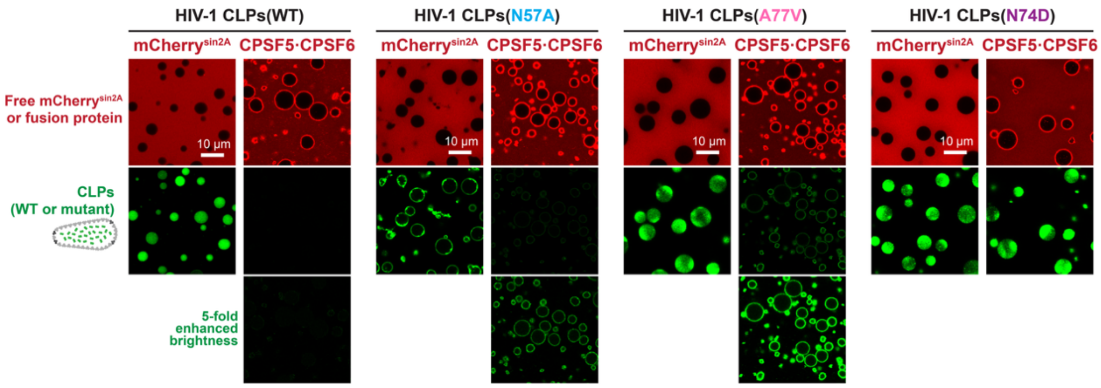

CLPs	Wildtype (WT)	N57A	A77V	N74D
FG phase-partitioning	+++	+	+++	+++
CPSF6 competition	+++	+	+	-

Supplementary Figure S2. Pocket mutations discriminate between GLFG repeat and CPSF6 binding.

Experiment compares wildtype CLPs with CLPs that carry ‘pocket mutations’ known to interfere with the CPSF6 interaction[1-3]. Capsids were allowed to partition in an GLFG phase, either directly or in the presence of the CPSF5·CPSF6 complex (at a 2.5:1 molar ratio to CA), as described in Fig.7a. Wildtype CLPs efficiently partitioned in the GLFG phase and this partitioning was fully blocked by CPSF6 (~0.2% residual signal). The N57A mutation impedes (but not fully abrogates) the interactions with CPSF6 and with barrier-forming FG repeats. The pentamer-only mutations (see Fig.7b) and the N74D exchange are most detrimental to the CPSF6 interaction, while causing no defect in FG phase-partitioning. A77V weakens interactions with CPSF6 but not with standard barrier-forming FG repeats. The lower tolerance to mutations indicates that the CPSF6 FG repeat docks into the pocket with more constraints and more contacts than a GLFG repeat unit of the FG phase.

Also note that the CPSF5-CPSF6 complex bound prominently to the outside of the FG phase. An explanation is that the proline-rich low complexity region (PR-LCR) of CPSF6 inserts into the FG phase but the FG-repulsive CPSF6 N-terminus prevents a full entry (see Supplementary Fig.3). The capsid does not co-localize with this surface-bound CPSF6 population. This points to a switch-like feature, namely that the PR-LCR (containing the CPSF6 FG motif) is not available for capsid-binding when partitioning into the FG phase and *vice versa*.

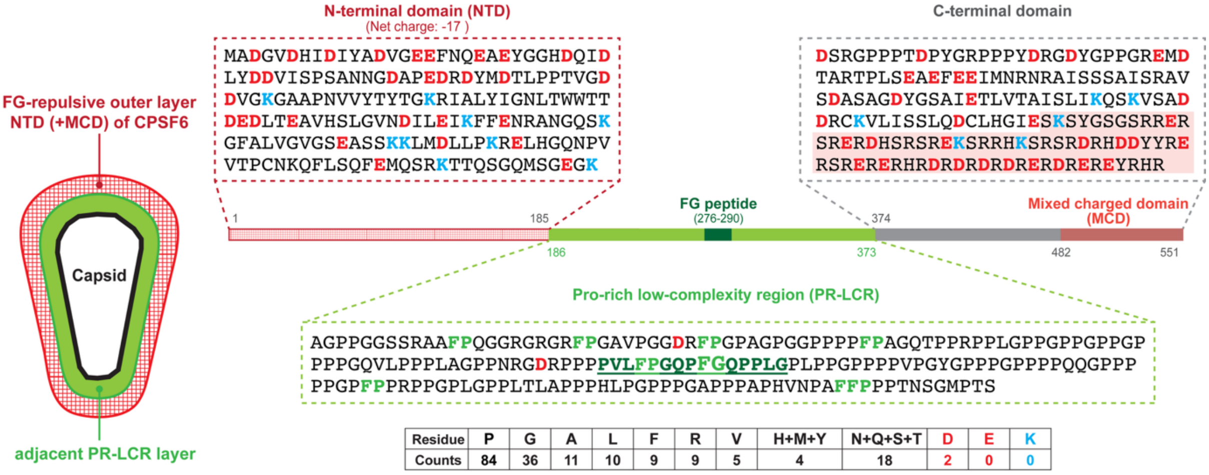

Residue	P	G	A	L	F	R	V	H+M+Y	N+Q+S+T	D	E	K
Counts	84	36	11	10	9	9	5	4	18	2	0	0

Supplementary Figure S3. Sequence features of CPSF6 and their relation to capsid release from the FG phase.

Sequence of CPSF6 (*UniProtKB:Q16630-1*), comprising three regions of different amino acid compositions:

- An N-terminal domain (NTD). It includes an RRM domain for heterotetramerization with CPSF5 (ref.[4]). Its large size (as a tetramer) and its strong negative charge should make it FG-repulsive.
- A proline-rich low complexity region (PR-LCR). It lacks FG-repulsive, charged residues (K, D, E) but is rather hydrophobic, whereby not only typical hydrophobic residues (like F, L, V and A) contribute but also proline, which accounts for 45% of the residues and provides (like valine) four aliphatic carbons for hydrophobic interactions. In terms of charge-depletion and hydrophobicity, the PR-LCR resembles an Nup98 FG domain. It should therefore also partition into an FG phase, explaining the prominent FG phase surface signal of the CPSF6 complex documented in Fig.7a and Supplementary Fig.S2. Since seven of the eight phenylalanines are in an FP and not in an FG context, the PR-LCR is, however, unlikely to recruit cellular NTRs. Nevertheless, it should readily phase-separate and engage in fuzzy hydrophobic interactions with the non-charged surface of the HIV capsid, once the embedded FG motif has docked into the N57 pocket of the capsid.
- A C-terminal ‘ mixed charged domain’ (MCD) that comprises the nuclear import signal of CPSF6 (ref.[1]). It is prone to phase-separation[5], probably through ionic and R-Y cation-π interactions. We assume that the MCD condensate is immiscible with the PR-LCR condensate or an FG phase. The phase separation propensity gets likely increased when phosphorylation of its SR motifs shifts its net charge to zero (isoelectric precipitation). Higher levels of phosphorylation would impede condensation and enhance FG-repulsion.

This architecture can explain why CPSF6 is so effective in antagonizing the partitioning of the HIV capsid into the FG phase and releasing it from the NPC into the nucleus: The PR-LCR forms a condensate layer around the capsid, thereby masking the N57 FG-binding pocket as well as the FG-attractive surface, while the negatively charged NTD remains excluded from the local condensate and forms an outer layer with FG-repulsive properties. This model attributes a second function to the very biased surface amino acid composition of the capsid, namely not only to allow a partitioning into the FG phase but also to recruit CPSF6 as a release factor. Note that while some aspects of this model still need some testing, others are already well supported (see e.g. ref.[5-9] and this study).

Supplementary Table S1. Sequences of FG domains used for FG phase experiments

FG domains	Sequence
**prf.GLFG$_{52x12}$**	**GLFG**GNTQPATG**GLFG**GNTQPATG**GLFG**GNTQPATG**GLFG**GNTQPATG**GLFG**GNTQPATG<br>**GLFG**GNTQPATG**GLFG**GNTQPATG**GLFG**GNTQPATG**GLFG**GNTQPATG**GLFG**GNTQPATG<br>**GLFG**GNTQPATG**GLFG**GNTQPATG**GLFG**GNTQPATG**GLFG**GNTQPATG**GLFG**GNTQPATG<br>**GLFG**GNTQPATG**GLFG**GNTQPATG**GLFG**GNTQPATG**GLFG**GNTQPATG**GLFG**GNTQPATG<br>**GLFG**GNTQPATG**GLFG**GNTQPATG**GLFG**GNTQPATG**GLFG**GNTQPATG**GLFG**GNTQPATG<br>**GLFG**GNTQPATG**GLFG**GNTQPATG**GLFG**GNTQPATG**GLFG**GNTQPATG**GLFG**GNTQPATG<br>**GLFG**GNTQPATG**GLFG**GNTQPATG**GLFG**GNTQPATG**GLFG**GNTQPATG**GLFG**GNTQPATG<br>**GLFG**GNTQPATG**GLFG**GNTQPATG**GLFG**GNTQPATG**GLFG**GNTQPATG**GLFG**GNTQPATG<br>**GLFG**GNTQPATG**GLFG**GNTQPATG**GLFG**GNTQPATG**GLFG**GNTQPATG**GLFG**GNTQPATG<br>**GLFG**GNTQPATG**GLFG**GNTQPATG**GLFG**GNTQPATG**GLFG**GNTQPATG**GLFG**GNTQPATG<br>**GLFG**GNTQPATG**GLFG**GNTQPATS
**SLFG$_{52x12}$**	**SLFG**NTGGAPAG**SLFG**NTQTQQGG**SLFG**QPQQTQGG**SLFG**QTGATTGG**SLFG**GATNTAPG<br>**SLFG**GGGGNPTG**SLFG**GNNNQQTG**SLFG**QGTTQTGG**SLFG**APQNNQGG**SLFG**GGTTTTGG<br>**SLFG**ANTQTGGG**SLFG**GPSQPTTA**SLFG**SNNPTTGG**SLFG**QPANTNNG**SLFG**GQTNNQAS<br>**SLFG**ANNQPPTN**SLFG**NNNKPQTA**SLFG**GATTTGNT**SLFG**GANNTGGG**SLFG**NNTNNPTG<br>**SLFG**ATNPAGGG**SLFG**GGATTGGG**SLFG**GGNTQTGG**SLFG**TANTTTAG**SLFG**GGNTQPQN<br>**SLFG**NNNTPATG**SLFG**QTNNAAPQ**SLFG**GTNNNAAS**SLFG**QKPASANGVLTKPNEKN<br>LCYAISNGTDFCIFELALTQRKLVKAGQLKPGAQAG**SLFG**QPAQNTQG**SLFG**GGGAATTP<br>**SLFG**GAQNNTTG**SLFG**GQNTQAGG**SLFG**APNNAAAT**SLFG**AGNANTQG**SLFG**AKPAATGG<br>**SLFG**QPAQTQAG**SLFG**NTAQPAGG**SLFG**GATTTPGG**SLFG**GNTAATGG**SLFG**GNTQGATG<br>**SLFG**GQQPNNQG**SLFG**NTNANTGG**SLFG**GATTTTGG**SLFG**GSTGATGG**SLFG**GASQPAAG<br>**SLFG**GAAPQQNS**SLFG**GATAGQTG**SLFG**GATQQQGG**SLFG**QTA
**FSFG**	**FSFG**NTGGAPAG**FSFG**NTQTQQGGSSSGQPQQTQGG**FSFG**QTGATTGG**FSFG**GATNTAPG<br>SSSGGGGGNPTG**FSFG**GNNNQQTG**FSFG**QGTTQTGGSSSGAPQNNQGG**FSFG**GGTTTTGG<br>**FSFG**ANTQTGGGSSSGGPSQPTTA**FSFG**SNNPTTGG**FSFG**QPANTNNGSSSGGQTNNQAS<br>**FSFG**ANNQPPTN**FSFG**NNNKPQTASSSGGATTTGNT**FSFG**GANNTGGG**FSFG**NNTNNPTG<br>SSSGATNPAGGG**FSFG**GGATTGGG**FSFG**GGNTQTGGSSSGTANTTTAG**FSFG**GGNTQPQN<br>**FSFG**NNNTPATGSSSGQTNNAAPQ**FSFG**GTNNNAAS**FSFG**QKPASANGVLTKPNEKN<br>LCYAISNGTDFCIFELALTQRKLVKAGQLKPGAQAGSSSGQPAQNTQG**FSFG**GGGAATTP<br>**FSFG**GAQNNTTGSSSGGQNTQAGG**FSFG**APNNAAAT**FSFG**AGNANTQGSSSGAKPAATGG<br>**FSFG**QPAQTQAG**FSFG**NTAQPAGGSSSGGATTTPGG**FSFG**GNTAATGG**FSFG**GNTQGATG<br>SSSGGQQPNNQG**FSFG**NTNANTGG**FSFG**GATTTTGGSSSGGSTGATGG**FSFG**GASQPAAG<br>**FSFG**GAAPQQNSSSSGGATAGQTG**FSFG**GATQQQGG**FSFG**QTASNPGGSSSGAANATTQP<br>**FSFG**GNNQAATS

Supplementary Table S2. Vectors to produce HIV-1 CA proteins for assembling CLPs

Plasmid	CA mutation(s)	C-terminal tag	Figure	Reference
pLF706	WT	-	1,3,4,5,6,7; ED4,5,6,7,8,10	[10]
pLF707	WT	EGFP	1,6,7	[10]
pLF974	WT	mScarlet-I3	6	This study
pLF721	N57A	-	1,3,4,5,6; ED4,5,7,10	This study
pLF731	N57A	EGFP	1,6	This study
pLF722	N74D	-	3; ED5	This study
pLF723	A77V	-	3; ED5	This study
pLF972	K182R	-	3; ED5	This study
pLF856	V86E	-	3,5; ED4,5	This study
pLF855	V86G	-	3; ED5	This study
pLF724	H87Q	-	3,5; ED4,5	This study
pLF859	H87E	-	3,5; ED4,5	This study
pLF858	H87G	-	3; ED5	This study
pLF862	A88E	-	3,5; ED4,5	This study
pLF861	A88G	-	3; ED5	This study
pLF725	G89V	-	3; ED5,8	This study
pLF726	P90A	-	3; ED5	This study
pLF867	I91E	-	3,5; ED4,5	This study
pLF866	I91G	-	3; ED5	This study
pLF727	A92E	-	3,4,5; ED4,5,6,7,10	This study
pLF869	A92G	-	3; ED5	This study
pLF728	G94D	-	3,4,5; ED4,5,7,10	This study
pLF873	M96E	-	3,5; ED4,5	This study
pLF872	M96G	-	3; ED5	This study
pLF875	R97K	-	3,5; ED5,6	This study
pLF729	E45A	-	3; ED5	This study
pLF838	G116A	-	3; ED5	This study
pLF966	R132K	-	3; ED5,6	This study
pLF967	L136M	-	3; ED5	This study
pLF969	L136E	-	ED6	This study
pLF970	R143A	-	3; ED5	This study
pLF971	R143K	-	ED6	This study
pLF958	Q9E	-	3,4,5; ED4,5,7,10	This study
pLF964	P123E	-	3,5; ED4,5	This study
pLF846	H87Q,A92E,G94D	-	4,5; ED4,7,10	This study
pLF842	N57A+A92E	-	4; ED4,7	This study
pLF844	N57A+G94D	-	4; ED4,7	This study
pLF956	N57A+A92E,G94D	-	4; ED4,7	This study
pLF848	N57A+H87Q,A92E,G94D	-	4,5; ED4,7,10	This study
pLF1018	Q9E+A92E	-	4,5; ED4,7,10	This study
pLF930	V86E,A88E,I91E,A92E	-	4,5,6; ED4,7,10	This study
pLF1010	V86E,A88E,I91E,A92E	EGFP	6	This study

All plasmids encode N-terminal His_{14}-bdSUMO tagged proteins for expression in *E. coli,* and all CA proteins carry a P1A mutation to allow for tag cleavage by bdSENP1.

Supplementary Table S3. Vectors to produce CA variants for assembling 40 nm spheres

Plasmid	CA mutation	C-terminal tag	Figure	Reference
pLF708	WT	-	1; ED3;S1	[10]
pLF709	WT	EGFP	1; ED3;S1	[10]
pLF741	N57A	-	1; ED3;S1	[10]
pLF751	N57A	EGFP	1; ED3;S1	[10]

All plasmids encode N-terminal His_{14}-bdSUMO tagged proteins for expression in *E. coli.* All CA proteins carry an P1A mutation to allow for tag cleavage, and the N21C, A22C mutations for disulfide-bridging CA protomers[11].

Supplementary Table S4. Vectors to produce CA variants for pentamer-only 20 nm spheres

Plasmid	CA mutation	C-terminal tag	Figure	Reference
pLF710	WT	-	1,4,7; ED1,4,7,8	This study
pLF711	WT	EGFP	1,4,7; ED1,4,7,8	This study
pLF761	N57A	-	1,4; ED4,7,8	This study
pLF771	N57A	EGFP	1,4; ED4,7,8	This study
pLF764	H87Q	-	4; ED4,7,8	This study
pLF774	H87Q	EGFP	4; ED4,7,8	This study
pLF765	G89V	-	ED8	This study
pLF775	G89V	EGFP	ED8	This study
pLF766	P90A	-	ED8	This study
pLF776	P90A	EGFP	ED8	This study
pLF767	A92E	-	4; ED4,7,8	This study
pLF777	A92E	EGFP	4; ED4,7,8	This study
pLF768	G94D	-	4; ED4,7,8	This study
pLF778	G94D	EGFP	4; ED4,7,8	This study
pLF990	Q9E	-	4; ED4,7,8	This study
pLF991	Q9E	EGFP	4; ED4,7,8	This study
pLF992	Q9E+A92E	-	4; ED4,7	This study
pLF993	Q9E+A92E	EGFP	4; ED4,7	This study
pLF1064	H87Q,A92E,G94D	-	4; ED4,7	This study
pLF1065	H87Q,A92E,G94D	EGFP	4; ED4,7	This study
pLF1066	V86E,A88E,I91E,A92E	-	4; ED4,7	This study
pLF1067	V86E,A88E,I91E,A92E	EGFP	4; ED4,7	This study

All plasmids encode N-terminal His_{14}-bdSUMO tagged proteins for expression in *E. coli.* All CA proteins carry an P1A mutation to allow for tag cleavage, and the G60A,G61P mutations that convert the capsid into the pentamer-only structure[12].

Supplementary Table S5. Plasmids used for other recombinant protein expression

Protein	Plasmid	Encoding	Figure(s)	Reference
**Probes**				
mCherry	pDG2442	His_{14}-bdSUMO-mCherry	1,3,4; ED1,3,6,8,10	[13]
EGFP	pSF1526	His_{14}-MBP-bdSUMO-EGFP	1; ED1	[13]
sinGFP4a	pDG2754	His_{14}-bdSUMO-sinGFP4a	3	[13]
2xMBP-Cys	pSNG133	His_{14}-bdSUMO-2xMBP-Cys	6	This study
Anti-Nup133 nanobody	pDG4439	His_{14}-NEDD8-xhNup133-Nb2t (+2Cys)	1,5	[14]
$mCherry^{sin2A}$	pDG2763	His_{14}-bdSUMO-$mCherry^{sin2A}$	7	This study
CPSF6-$mCherry^{sin2A}$	pDG5515	2xZpA963-bdSUMO-CPSF6-$mCherry^{sin2A}$-His_8	7	This study
$CPSF6_{(FG>GG)}$-$mCherry^{sin2A}$	pLF1275	2xZpA963-bdSUMO-$CPSF6_{(FG>GG)}$-$mCherry^{sin2A}$-His_8	7	This study
CPSF7-$mCherry^{sin2A}$	pDG5521	2xZpA963-bdSUMO-CPSF7-$mCherry^{sin2A}$-His_8	7	This study
CPSF5	pDG5263	2xZpA963-NEDD8-CPSF5	7	This study
**FG domains**				
prf.$GLFG_{52x12}$	pSNG57	His_{18}-$GLFG_{52x12}$	1,3,4,7; ED3,5,6,7,10	[15]
$SLFG_{52x12}$	pSNG73	His_{18}-$SLFG_{52x12}$	ED1;S1	[16]
$FSFG_{52x12}$	pSNG150	His_{18}-FSFG domain	ED1,8;S1	[16]

Supplementary Table S6. Sequence identifiers for Nup358 from various species

Species	Classification	Sequence identifier
Human (*Homo sapiens*)	Class: Mammalia, Order: Primates	UniProtKB/Swiss-Prot: P49792
Mouse (*Mus musculus*)	Class: Mammalia, Order: Rodentia	UniProtKB/Swiss-Prot: Q9ERU9
Chicken (*Gallus gallus*)	Class: Aves, Order: Galliformes	GenBank: XP_040513539.1
Snake (*Python bivittatus*)	Class: Reptilia, Order: Squamata	GenBank: XP_007427690.1
Fish (*Danio rerio*)	Class: Actinopterygii, Order: Cypriniformes,	GenBank: XP_021334470.1
Frog (*Xenopus laevis*)	Class: Amphibia, Order: Anura	GenBank: XP_041438290.1
Frog (*Xenopus tropicalis*)	Class: Amphibia, Order: Anura	GenBank: AAI57560.1
Frog (*Rana temporaria*)	Class: Amphibia, Order: Anura	GenBank: XP_040192403.1
Toad (*Bufo bufo*)	Class: Amphibia, Order: Anura	GenBank: XP_040281002.1
Toad (*Bufo gargarizans*)	Class: Amphibia, Order: Anura	GenBank: XP_044139501.1
Toad (*Bombina bombina*)	Class: Amphibia, Order: Anura	GenBank: XP_053561416.1
Salamander (*Ambystoma mexicanum*)	Class: Amphibia, Order: Urodela	GenBank: XP_069492978.1
Salamander (*Pleurodeles waltl*)	Class: Amphibia, Order: Urodela	GenBank: XP_069060581.1
Caecilian (*Geotrypetes seraphini*)	Class: Amphibia, Order: Gymnophiona	GenBank: XP_033804751.1
Caecilian (*Rhinatrema bivittatum*)	Class: Amphibia, Order: Gymnophiona	GenBank: XP_029460448.1

Supplementary Table S7. Sequence identifiers for CA proteins from various lentiviruses

Lentivirus	Subtype	Isolate	Host	Sequence identifier
**HIV-1**				
HIV-1:M:B (KC005)	M, B	KC005	Human (*Homo sapiens*)	GenBank: CBI62787.1
HIV-1:M:B (NY5)	M, B	NY5	Human (*Homo sapiens*)	GenBank: AAB04036.1
HIV-1:M:A (U455)	M, A	U455	Human (*Homo sapiens*)	GenBank: AAA75018.1
HIV-1:M:C (92BR025)	M, C	92BR025	Human (*Homo sapiens*)	UniProtKB/Swiss-Prot: O12158.2
HIV-1:N (YBF30)	N	YBF30	Human (*Homo sapiens*)	UniProtKB/Swiss-Prot: O91079.3
HIV-1:O (ANT70)	O	ANT70	Human (*Homo sapiens*)	UniProtKB/Swiss-Prot: Q77372.3
HIV-1:P (U14788)	P	U14788	Human (Homo sapiens)	GenBank: ADR03143.1
**HIV-2**				
HIV-2:A (ROD)	A	ROD	Human (*Homo sapiens*)	UniProtKB/Swiss-Prot: P04590.3
HIV-2:B (D205,7)	B	D205,7	Human (*Homo sapiens*)	UniProtKB/Swiss-Prot: P15832.3
HIV-2 (CAM2)	other	CAM2	Human (*Homo sapiens*)	UniProtKB/Swiss-Prot: P24106.3
**SIV**				
SIVcpz (MB66)		MB66	Chimpanzee (*Pan troglodytes troglodytes*)	UniProtKB/Swiss-Prot: Q1A268.3
SIVcpz (EK505)		EK505	Chimpanzee (*Pan troglodytes troglodytes*)	UniProtKB/Swiss-Prot: Q1A249.3
SIVcpz (GAB1)		GAB1	Chimpanzee (*Pan troglodytes troglodytes*)	UniProtKB/Swiss-Prot: P17282.1
SIVgor (BPID1)		BPID1	Gorilla (*Gorilla gorilla gorilla*)	GenBank: AJT49601.1
SIVgor (BQID2)		BQID2	Gorilla (*Gorilla gorilla gorilla*)	GenBank: AJT49619.1
SIVmac239		SIVmac239	Rhesus macaque (*Macaca mulatta*)	GenBank: AQY10339.1
SIVstm		STM	Stump-tailed macaque (*Macaca arctoides*)	UniProtKB/Swiss-Prot: P31634.1
SIVagm (sab-1)		sab-1	Green monkey (*Chlorocebus sabaeus*)	GenBank: AAA21504.1
SIVagm (tan-1)		tan-1	Tantalus monkey (*Chlorocebus tantalus*)	GenBank: AAC57051.1
SIVmnd-2		SIVmnd-2	Mandrill (*Mandrillus sphinx*)	GenBank: AAM08705.2
SIVmnd (GB1)		GB1	Mandrill (*Mandrillus sphinx*)	UniProtKB/Swiss-Prot: P22381.1
SIVwrc		WRC	Red colobus monkey (*Piliocolobus badius temminckii*)	GenBank: CAP72503.1
SIVolc		OLC	Olive colobus monkey (*Procolobus verus*)	GenBank: CAQ60124.1

Supplementary References

1. Lee, K. et al. Flexible use of nuclear import pathways by HIV-1. *Cell Host Microbe* **7**, 221-233 (2010).
2. Price, A. J. et al. CPSF6 defines a conserved capsid interface that modulates HIV-1 replication. *PLoS Pathog* **8**, e1002896 (2012).
3. Matreyek, K. A., Yücel, S. S., Li, X. & Engelman, A. Nucleoporin NUP153 phenylalanine-glycine motifs engage a common binding pocket within the HIV-1 capsid protein to mediate lentiviral infectivity. *PLoS Pathog* **9**, e1003693 (2013).
4. Yang, Q., Coseno, M., Gilmartin, G. M. & Doublié, S. Crystal structure of a human cleavage factor CFI(m)25/CFI(m)68/RNA complex provides an insight into poly(A) site recognition and RNA looping. *Structure* **19**, 368-377 (2011).
5. Jang, S. et al. HIV-1 usurps mixed-charge domain-dependent CPSF6 phase separation for higher-order capsid binding, nuclear entry and viral DNA integration. *Nucleic Acids Res* **52**, 11060-11082 (2024).
6. Bejarano, D. A. et al. HIV-1 nuclear import in macrophages is regulated by CPSF6-capsid interactions at the nuclear pore complex. *Elife* **8**, e41800 (2019).
7. Francis, A. C. et al. HIV-1 replication complexes accumulate in nuclear speckles and integrate into speckle-associated genomic domains. *Nat Commun* **11**, 3505 (2020).
8. Wei, G. et al. Prion-like low complexity regions enable avid virus-host interactions during HIV-1 infection. *Nat Commun* **13**, 5879 (2022).
9. Ay, S. & Di Nunzio, F. HIV-Induced CPSF6 Condensates. *J Mol Biol* **435**, 168094 (2023).
10. Fu, L. et al. HIV-1 capsids enter the FG phase of nuclear pores like a transport receptor. *Nature* **626**, 843-851 (2024).
11. Pornillos, O., Ganser-Pornillos, B. K., Banumathi, S., Hua, Y. & Yeager, M. Disulfide bond stabilization of the hexameric capsomer of human immunodeficiency virus. *J Mol Biol* **401**, 985-995 (2010).
12. Schirra, R. T. et al. A molecular switch modulates assembly and host factor binding of the HIV-1 capsid. *Nat Struct Mol Biol* **30**, 383-390 (2023).
13. Frey, S. et al. Surface properties determining passage rates of proteins through nuclear pores. *Cell* **174**, 202-217.e9 (2018).
14. Solà Colom, M. et al. A checkpoint function for Nup98 in nuclear pore formation suggested by novel inhibitory nanobodies. *EMBO J* **43**, 2198-2232 (2024).
15. Ng, S. C., Güttler, T. & Görlich, D. Recapitulation of selective nuclear import and export with a perfectly repeated 12mer GLFG peptide. *Nat Commun* **12**, 4047 (2021).
16. Ng, S. C. et al. Barrier properties of Nup98 FG phases ruled by FG motif identity and inter-FG spacer length. *Nat Commun* **14**, 747 (2023).
